# Finite Adaptation and Multistep Moves in the Metropolis-Hastings Algorithm for Variable Selection in Genome-Wide Association Analysis

**DOI:** 10.1371/journal.pone.0049445

**Published:** 2012-11-15

**Authors:** Tomi Peltola, Pekka Marttinen, Aki Vehtari

**Affiliations:** 1 Department of Biomedical Engineering and Computational Science, Aalto University, Espoo, Finland; 2 Department of Information and Computer Science, Aalto University, Espoo, Finland; Queen’s University Belfast, United Kingdom

## Abstract

High-dimensional datasets with large amounts of redundant information are nowadays available for *hypothesis-free* exploration of scientific questions. A particular case is genome-wide association analysis, where variations in the genome are searched for effects on disease or other traits. Bayesian variable selection has been demonstrated as a possible analysis approach, which can account for the multifactorial nature of the genetic effects in a linear regression model.

Yet, the computation presents a challenge and application to large-scale data is not routine. Here, we study aspects of the computation using the Metropolis-Hastings algorithm for the variable selection: finite adaptation of the proposal distributions, multistep moves for changing the inclusion state of multiple variables in a single proposal and multistep move size adaptation. We also experiment with a delayed rejection step for the multistep moves. Results on simulated and real data show increase in the sampling efficiency. We also demonstrate that with application specific proposals, the approach can overcome a specific mixing problem in real data with 3822 individuals and 1,051,811 single nucleotide polymorphisms and uncover a variant pair with synergistic effect on the studied trait. Moreover, we illustrate multimodality in the real dataset related to a restrictive prior distribution on the genetic effect sizes and advocate a more flexible alternative.

## Introduction

The progress in high-throughput measurement technologies has allowed application specialists to gather extensive datasets with often large amounts of redundant information for the addressed scientific question. This is particularly true in (human) genetics, where it has become cost-effective to measure individual genetic variation at the scale of millions of polymorphic sites in the DNA. Numerous genome-wide association studies (GWAS) have been published during the last decade linking the genetic variation to disease and other traits [1].

However, such data analysis is not without problems. The primary association analyses in GWAS are mainly conducted by testing each polymorphic site, usually single nucleotide polymorphism (SNP), for association independently and then correcting for multiple hypothesis testing. This simplification is computationally convenient, but does not acknowledge the hypothesis of multifactorial genetic background for many common diseases and traits. Alternatives, which consider all of the genetic variants simultaneously, include penalized multivariate regression and variable selection methods (e.g., [2], [3]).

In this work, we focus on the computation of the Bayesian linear regression model with variable selection using Markov chain Monte Carlo (MCMC) methods. The variable selection is a natural fit for the main task in GWAS of searching for the genetic variants showing association to a phenotype of interest, and such models have been recently applied successfully to various sizes of genetic datasets including full GWAS scale [3], [4]. These models introduce latent binary indicator variables 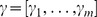 to specify the inclusion status of each genetic variant (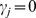 or 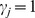) in the regression model. The expected sparsity is encoded into the prior distribution of the indicators. The relevant posterior quantities are then obtained through model averaging (where model refers to a configuration of the indicator vector 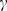). However, the computation can be challenging as the Markov chains may suffer from long autocorrelation.

A general approach to the variable selection in this framework is the Metropolis-Hastings algorithm (MH) [5], [6], where to generate samples from the posterior distribution, changes to the state of the indicator vector 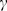 are proposed from a proposal distribution 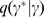 and then accepted as the new state or rejected (duplicating the previous state in the MCMC chain) according to the MH acceptance probability:
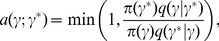
(1)where 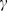 and 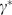 are the current and the proposed state and 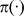 is the posterior probability.

Here, we study the following ideas in formulating the proposal distribution *q*: 1) finite adaptation of the proposal distributions for adding and removing variables from the model, 2) adding and removing multiple variables in a single proposal (multistep move) with finite adaptation of the move size (the number of additions/removals proposed) and 3) delayed rejection [7], [8], which re-utilizes some of the computations leading to a rejected proposal in making a second proposal from a larger set of states. The resulting sampling algorithms are studied on simulated data and a real GWAS dataset with nearly four thousand individuals and over one million SNPs (analyzed previously in [4]) with a focus on the efficiency of the sampling. We further describe additional proposals tailored to the genetic data, which help against specific convergence and mixing problems encountered in the real data, and demonstrate in the real data that a prior, which is flexible to having few large effect sizes among many small, may be desirable.

The motivation for adapting the proposal distributions stems from the *small n, large p* property of the data with most of the *p* variables being irrelevant. Proposing updates to 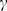 uniformly from the large set of variables may waste lots of computation time on rejecting poor proposals and be slow to find high posterior probability models. Here, the marginal inclusion probabilities of the variables will be used to form the proposal distributions, which are adapted during an initial phase in the sampling before collecting samples for posterior inference (finite adaptation). This is similar to the (full) adaptive sampler of Nott and Kohn [9]. The Bayesian adaptive sampling algorithm (BAS) [10] also uses the marginal inclusion probabilities for sampling. It differs from the above mentioned in that it samples models without replacement (and is not an MCMC method). Our previous work [4] included finite adaptation of the proposal distribution for (single) additions, while Guan and Stephens [3] have used statistics from single variable analyses to form the proposal distribution for additions. The latter two articles do not study the efficiency of the samplers.

Multistep moves have been used in GWAS setting by Guan and Stephens [3], but they provide little details beyond the mention of generating them as combinations of single additions and removals. As the multistep proposals for updating 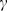 do not come from a uniform distribution, some care is required in formulating *q* in a proper way. Here, the sequential Metropolis-Hastings proposal framework of Storvik [11] will be utilized to provide theoretical validity of the resulting Markov chain. Lamnisos et al. [12] discuss the adaptation of the move size in multistep moves with uniform proposal distribution for variable inclusion updates. They use acceptance rate coercion to adapt the move size proposal distribution, which relies on the knowledge or estimate of optimal acceptance rate. An alternative approach is provided by Pasarica and Gelman [13], who maximize the expected jump distance of the Markov chain (corresponding to minimizing the first autocorrelation), and is here introduced in the variable selection context. This has the advantage of not relying on the availability of the knowledge of the optimal acceptance rate.

We also experiment with a novel delayed rejection step, which re-utilizes some of the computations leading to a rejected multistep proposal. In the delayed rejection algorithm if the first proposal is rejected, another proposal may be made. Here, assuming a *k*-step proposal, which is rejected, the full set of posterior probabilities of the 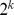 models available from changes to the inclusion status of the *k* variables can be computed using relatively cheap updates to the likelihood of the full model (particularly, the Cholesky decomposition of the covariance matrix), which is available fully or in part from the rejected proposal. A second proposal is then made from this set of models utilizing the computed posterior probabilities.

An open source C++ implementation of the samplers presented here is available at http://becs.aalto.fi/en/research/bayes/bmagwa/ and https://github.com/to-mi/. It has been specifically developed for GWA analysis allowing for fast and memory-efficient handling of large datasets.

## Methods

### Model

The model mapping from genotypes (values of the explanatory variables) to a phenotype (the target variable) is briefly introduced here. This is essentially the same as in our previous work [4], except here we consider only additive formulation for the genetic effects and introduce a more flexible prior for the variance of the effect sizes. For similar alternatives, see, for example, references [3], [14], [15].

A linear regression model is used:
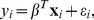
(2)where 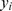, 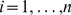, are the values of the phenotype for *n* individuals, 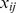, 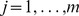, are genotypes for *m* SNPs and 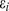 are residuals, which are assumed to follow a zero-mean normal distribution with variance 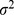: 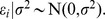


To facilitate variable selection, binary variables 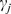 are used to indicate the presence of effect 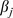. That is, for 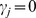, 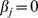 and for 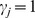, 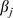 may be non-zero. The prior structure for the model parameters is:
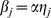


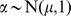


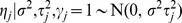


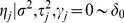
(3)

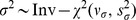


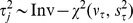


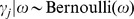


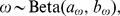
where 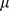 is the mean of the prior for 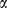, 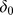 is the Dirac delta function at zero and 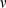 and 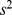 refer to the degrees of freedom and scale parameters of the (scaled) 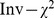 distributions. 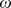 is the prior probability of 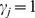 with prior expectation 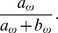 *j* runs from 1 to *m*.

**Figure 1:**
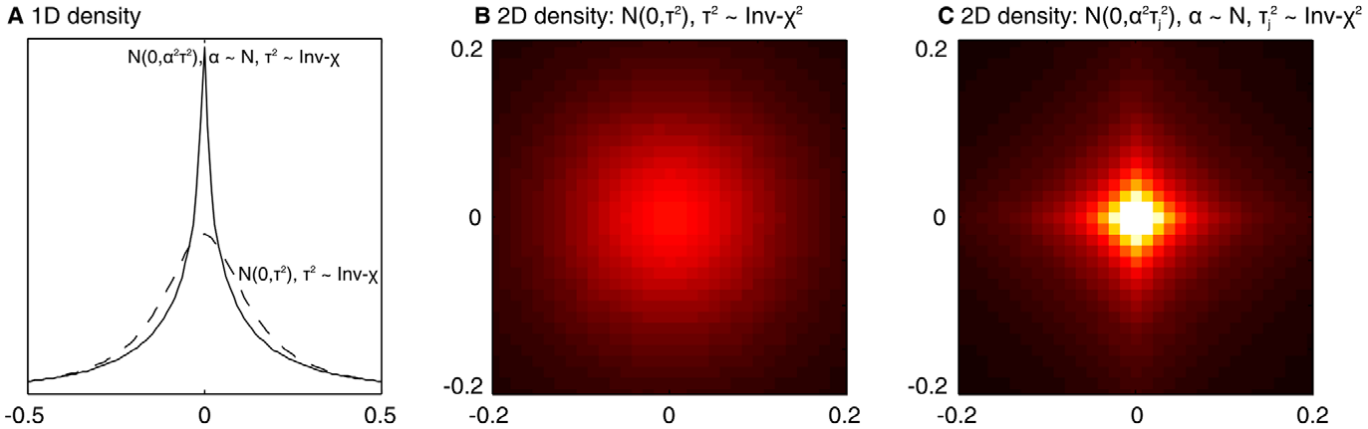
Illustration of the effect size prior. A. 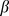 prior density with 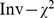 and noncentral-F (from 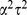 with 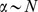 and 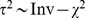) distributions for the variance. The former yields a *t*-distribution. The latter is more spiked. Both have heavier tails than normal distribution. Panels B and C show the comparison in two dimensions (pseudo-colored histograms with dark as low and bright as high values). In the former the two 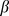
s share the 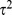 parameter, whereas they have independent 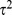 parameters in the latter. The plots were constructed from 50 million samples with fixed prior parameters 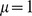, 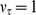 and 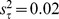 (and assuming 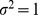).

The prior of the effect sizes, 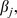 is a zero-mean normal distribution with a noncentral-F prior for variance [16]. This is more flexible than the 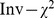 distribution for variance, which we have used previously [4], but is still convenient to sample. Here, 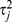 are also variable specific (previously a single parameter was shared), which places more mass on 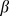 with few large effects among many small ones and seems appropriate in the lipoprotein cholesterol analyses. Figure 1 illustrates the 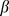 prior. The prior for 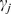 induces sparsity into the model. When available, published analyses may be used to guide the selection of the prior parameters (proportion of variance explained for 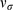 and 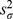; effect sizes for 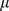, 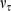 and 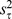; number of associations for 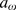 and 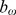; see [4]).

### Computation

The overview of the Markov chain Monte Carlo algorithm used to sample from the posterior distribution of the parameters of the above model is given here briefly, before focusing on the specifics of the sampling of 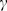.

The linear model given 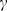, 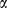 and 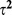 has conjugate structure allowing integration over 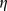 and 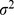 analytically, which is utilized below in the third step. 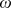 is integrated out analytically and not sampled. The following Gibbs sampling scheme is used for the remaining parameters (see Text S1 for details on the conditional distributions):

1. Sample 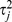
s given the other parameters from scaled inverse-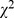 distributions.
2. Sample 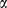 given the other parameters from a normal distribution.
3. Sample 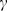 given 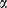 and 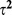 with a Metropolis-Hastings step.
4. Sample 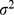 given 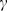, 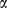 and 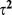 from a scaled inverse-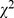 distribution.
5. Sample 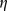 given the other parameters from a normal distribution.

The last three steps are a factorized draw from 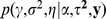. Additionally (if 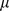 is not zero), a deterministic Metropolis proposal to flip the signs of 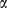 and 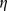 is included to avoid getting 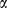 stuck into negative or positive values (note that this move has no effect on the signs of 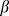
s). Steps 1, 2, 4 and 5 are done only every tenth (or hundredth for alternative algorithms) iteration in our experiments, as the sampling of 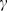 in the third step is often the most challenging one.

For posterior inference, the Rao-Blackwellization method of Guan and Stephens [3] is used to estimate the posterior association probabilities 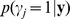, denoted 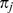 for short (see also [4]). It essentially works by periodically computing single variable linear regressions for each variable against the residual of the current linear regression model (at some sampled state 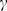) and updating the estimates 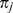 accordingly.

### Algorithms for Variable Inclusion Updates

Three algorithms will be described for the Metropolis-Hastings step (MH) step, which is used to update 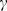 in the third sampling step:

1. Single step (SS) algorithm, which proposes a change to a single 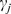 in each iteration.
2. Multistep (MS) algorithm, which proposes multiple changes to 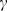 in each iteration.
3. Multistep algorithm with delayed rejection (MS-DR).

The proposals are formed in two main steps: 1) move size (number of changes) proposal and 2) sequential proposal of the variables to update (add to or remove from the model). The proposal is then accepted or rejected according to the MH acceptance probability. The single step algorithm always chooses move size of one.

The parameters of the proposal distribution may be adapted during an initial phase in the sampling (giving a total of six different samplers; three adaptive and three non-adaptive). The parameters are then fixed before collecting posterior samples (finite adaptation). Non-adaptive algorithms employ uniform distribution to generate the proposals (expect that move size adaptation is allowed here for all multistep samplers to avoid trial-and-error in finding a good proposal distribution). Brief descriptions of the sampling and adaptation are given below. Details are given in Text S1.

#### Move size proposal

The proposal distribution 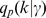 for move size *k* should preferably have only a single parameter in order to make adaptation simple. We have chosen to use a truncated geometric distribution, where the parameter 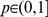 governs the shape of the distribution. Geometric distribution is more conservative a choice than, for example, the binomial distribution in the regard that the move size 1 is always the single most probable value. We use a fixed value equal to 20 as the truncation point, while *p* is adapted. For adaptation we use expected jump distance optimization described below (for an alternative, see [12]).

Pasarica and Gelman [13] optimize the expected squared jump distance in a Gaussian proposal distribution, the motivation of which stems from the formula 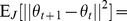 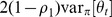, where *J* is the kernel of the Markov chain with some optimizable parameter, 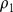 the lag one autocorrelation and 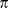 the stationary distribution of the sampled parameter 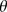. Thus, maximizing the expectation corresponds to minimizing the first autocorrelation, which may lessen the dependencies between consecutive samples. Using the approach in variable selection context for move size proposals is straightforward and does not rely on assumptions about optimal acceptance rate for the problem at hand.

In order to derive the connection of the expected squared jump distance and lag one autocorrelation in the present context, the mean and variance of 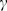 and its lag one autocorrelation (times variance) for the Markov chain are defined as
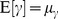


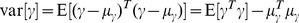
(4)

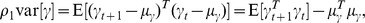
where the variance and covariance are taken as sums of the variances and covariances of the individual components. With these at hand, the expected squared jump distance can be seen to be 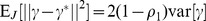. We note that for vectors of binary values the squared distance is equal to the Hamming distance^1^. ^1^ 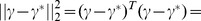 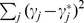 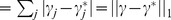 as 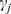 can take values 0 and 1.

Pasarica and Gelman [13] suggest using covariance norm in the case of multidimensional targets, but estimating the covariance matrix would be difficult here.

The objective function to maximize with regard to the parameter *p* is.
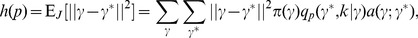
(5)where 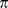 is the stationary distribution and *a* is the acceptance probability of a move from 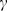 to 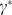. The acceptance probability will be independent of *p* as the corresponding factors cancel in the MH ratio. Samples from the adaptive phase of our MCMC algorithm are used in the multiple importance sampling estimator of Pasarica and Gelman [13] to evaluate this objective (for details, see Text S1).

#### Sequential proposal for variable inclusion updates

Given the move size, additions and removals are proposed in a sequence with probability 0.5 (unless there are no variables to add or remove). Denoting the sequence of proposed changes using auxiliary variables 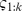, the proposal distribution can be written as a product 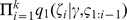, where 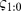 is taken as the empty sequence. The individual proposal distributions 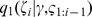 for selecting the variables to add or remove are formed according to the estimates of the marginal inclusion probabilities 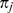 of the variables, which are continuously updated during the adaptive phase of sampling using the Rao-Blackwellization method [3]. The proposals for variables to add are generated by sampling variables proportional to the estimated inclusion probabilities (with bounding away from zero using a preset minimum value) unless the variable has already been proposed to be added in this round. Variables to remove are sampled identically except for the sampling probabilities being proportional to 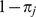.

Alternatively, the usual MCMC estimates of 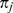 could be used in the adaptation with a smaller computational cost than the Rao-Blackwellization, but the latter provides more robust estimates especially at the beginning of the sampling and when the number of variables is large.

The above sampling scheme is here cast into the form of Storvik [11] (with some differences in notation) to write the acceptance probability and show the validity of the scheme. The full proposal distribution is written as
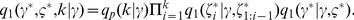
(6)


**Figure 2:**
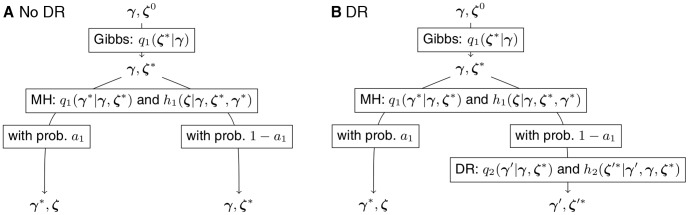
Flow diagram of the proposal. A. View of the full move as a Gibbs step followed by a Metropolis-Hastings (MH) step. B. Delayed rejection (DR): a second proposal may be done when the first proposal is rejected. Since the DR proposal is constructed here such that it is always accepted, there is no further branching after the second proposal. 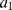 is the acceptance probability of the MH step. 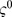 refers to an old value of the auxiliary variable, which is irrelevant.

In this, 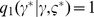 if 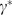 is the model derived from 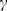 with the operations specified by 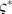 and zero otherwise. In order to be able to calculate the Metropolis-Hastings acceptance probability, the sequence of auxiliary variables related to the reverse proposal must be specified. To this end, a distribution 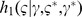 is introduced. The distribution 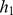 places unit probability to a single sequence of auxiliary variables which is obtained from 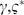 and 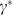 using a specific deterministic procedure (see below). Given these distributions, the acceptance probability for the proposal is
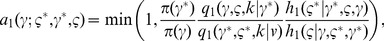
(7)which, according to Proposition 2 of Storvik [11], leads to samples from the correct target distribution with proper convergence and ergodicity results when the Markov chain is irreducible. For some insight, the move may be viewed as an iteration of an MCMC for sampling from the joint distribution 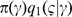, which has the correct marginal for 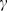. The iteration consists of a Gibbs step updating 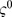 to 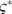, followed by a Metropolis-Hastings step with the specified acceptance probability during which 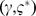 is proposed to be replaced by 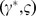. This is illustrated in Figure 2A.

Regarding 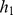, a simple approach would be to take 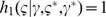, if 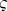 is the reverse of 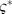 (i.e., additions become removals with the sampling order reversed and vice versa) and zero otherwise. However, to be consistent with the delayed rejection implementation, a slightly more complex deterministic procedure is chosen here (see Text S1).

An alternative to introducing the sampling order to the acceptance probability would be to sum over the different orderings of 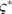. See Text S1 for a comment on this.

#### Example 1

Here we illustrate the notation and behavior of the sampling algorithm using a concrete, albeit overly simplistic, example. Suppose the total number of SNPs in data is equal to 5 and let the current state of the algorithm be 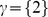, i.e., the second SNP is currently included in the model. Note that here, for shortcut, we represent 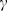, actually a vector of indicators, as a set of non-zero indicators. The sampling then proceeds as follows. 1) The number of updates, *k*, is drawn. Suppose that 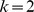 is selected. 2) The type of update (addition/removal) and the SNP involved is determined in turn for each update. Suppose this results in the sequence of auxiliary variables 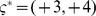, meaning that SNPs 3 and 4 are proposed to be added to the model. This, in turn, fixes the proposed new state to 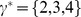. Furthermore, this fixes the sequence of auxiliary variables in the reverse proposal to 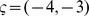. Recall that 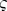 is determined using the distribution 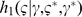, which places a unit mass on a single sequence of auxiliary variables using the deterministic procedure, as described earlier. Also note that applying 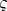 to 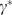 would change the state back to 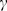 again, as required. 3) Finally, the acceptance probability specified in Equation 7 is used to decide whether to change the current state from 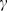 to 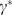.

#### Delayed rejection

Delayed rejection [7], [8] builds on the result of Peskun [17], which states that given two transition probability matrices of Markov chains, the one with greater off-diagonal elements has lower asymptotic variance for the MCMC estimate of an expectation of a function. Whereas the MH sampling algorithm replicates the old state on rejection and proceeds to the next iteration, the delayed rejection algorithm makes a second proposal (and possibly more), which is then considered for acceptance. The acceptance probability is constructed to preserve the reversibility of the Markov chain. The algorithm can only increase the off-diagonal mass in the transition matrix as the acceptance probability of the first proposal is not affected.

An essential feature of delayed rejection is that the second proposal may depend on the first. Here, this is taken advantage of by re-utilizing the computations performed for the first proposal. Note that the time complexity of computing the likelihood after the first proposal has been made is dominated by the updates to the Cholesky decomposition of the covariance matrix of the predictors (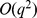) and computation of the covariances when variables are added (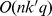 with *q* the number of variables in the model and 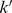 the number of new variables). Now, following a proposal from 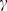 to 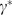 through auxiliary variable 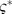 which is to be rejected, another proposal is made instead. The second proposal is sampled from the set of models, which can be constructed by flipping elements of 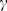 with the flips restricted to the variables indicated by 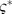. There are 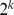 such models, where *k* is the move size of the first proposal. Given the Cholesky decomposition of the largest model, computation of the posterior probabilities of the whole set of models may be done in 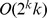 (see Text S1 for more details). This overhead is often small compared to making a completely new proposal, when *q* or *n* are large relative to *k* and allows the sampling to use the knowledge of the posterior probabilities of 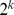 models.

The acceptance probability of the second proposal preserving reversibility is given by:
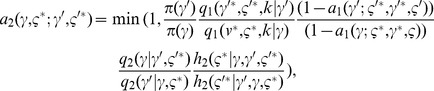
(8)where items related to the second proposal are marked with 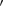 and the ratio for 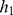 is dropped to simplify notation. Note that 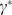 and 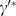, which are the first proposals in the forward and backward routes, are not constrained to be equal. 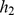 will be chosen to be deterministic similarly to 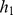. We have constructed the proposal distributions such that the second proposal is always accepted. The notation and the course of action of the delayed rejection are illustrated in Figure 2B and through the following example. Further details are provided in the Text S1.

#### Example 2

Here we illustrate the delayed rejection part of the sampling algorithm by continuing from Example 1 and assuming that the suggested move from 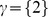 to 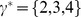 was rejected. Recall also that the sequence of auxiliary variables related to the first proposal was 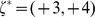. Thus, in the first proposal, SNPs 3 and 4 were proposed to be added to the model. The big picture here is that starting from the rejected first proposal we make a second proposal. To calculate the Metropolis-Hastings acceptance probability of this *two-step forward proposal*, a corresponding *two-step backward proposal* must be specified. In our approach, the backward route is fixed deterministically such that the second step of the forward proposal is always accepted.

The delayed rejection part of the algorithm proceeds by sampling the second (forward) proposal from the set of all models which can be reached from the initial state 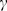 by applying any subset of operations in 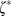. Here, we will denote this set of models by 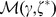. Consequently, 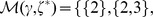 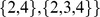. The models are sampled from 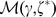 using a distribution 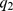 which is selected such that it cancels the terms 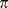, 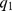 and 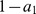 in the numerator of the acceptance probability given in Equation 8. Suppose the model proposed is 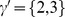. Note that no auxiliary variables are related to this second proposal, as the model itself is sampled directly.

Now, the two-step backward proposal is determined as follows: first, auxiliary variables related to the *first step in the backward proposal* are deterministically set to 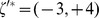 corresponding to proposing the model 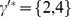. This follows, because it is required that 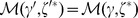, i.e. that the second proposals in both forward and backward moves are sampled from the same set of models. To calculate the acceptance probability of this first step in the backward proposal, the reversed sequence 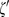 of auxiliary variables is required similarly to the first step in the forward proposal (see Example 1), yielding 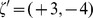.

After rejection of the first step in the backward proposal, the second step must change the state back to the original model 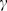. With these specifications at hand, the acceptance probability of the second step in the forward proposal can be evaluated using Equation 8, and is found to be equal to unity. In summary, the only variables that were sampled during the whole MCMC step are: 1) the sequence of auxiliary variables sampled in the first step of the forward proposal, 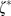, and 2) the model sampled in the second step of the forward proposal, 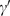. All other variables required when calculating the acceptance probability follow deterministically from these two along with the initial state 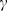.

#### Additional moves for SNP data

Two additional moves are introduced specifically for genetic data, where the variables can be ordered linearly (corresponding to their locations in the genome) and neighboring variables may have block-like correlation structure (linkage disequilibrium), which may complicate the mixing of the Markov chain.

The first move type proceeds by selecting one variable in the model (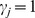) randomly to be swapped with a variable which is located in its neighborhood (defined by a cutoff in the distance of the linear indices) and is not in the model (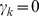). A similar move is also considered by Guan and Stephens [3]. The second move type begins identically by randomly selecting one variable in the model (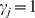). Then, an update to a randomly selected neighboring variable (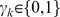) is proposed. For both of these move types, multiple updates of the same type may be incorporated into a single proposal. Further, delayed rejection is allowed for the latter move type (i.e., we allow reverting some of the proposed updates in a multistep proposal similarly to the delayed rejection described above, but with simpler acceptance probability as the updates are proposed from a uniform distribution).

In our implementation each of the additional move types is proposed with probability 0.15 and the main 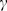 update with probability 0.7. The move size in the additional move types is determined using the truncated geometric distribution with a fixed parameter (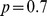 for the first additional move type, 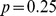 for the second).

### Comparison Algorithms

The algorithms introduced above are compared to random scan versions of Kohn-Smith-Chan (KSC) [18] and Nott-Kohn (NK) [9] sampling algorithms. A proposal of both algorithms first selects *k* variables in random for consideration (here *k* is fixed to 1, 5 or 10) and propose a new model 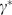 from the set of 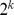 models available by flipping the inclusions of the selected variables. The KSC algorithm makes this proposal with the proposal probabilities proportional to the prior probabilities of the models. The NK algorithm uses an adaptive distribution for the proposal. Here, we restrict the adaptation to be finite and use the same kind of tuning as in the proposed adaptive algorithms. The proposal distribution is taken as a independent combination of the adapted marginal inclusion probabilities: 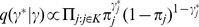 with *K* representing the set of the selected *k* variables. See Text S1 for more details and for a note on the similarity of the NK and the proposed algorithm.

### Ethics Statement

Human data was not collected primarily for this article and was analyzed here anonymously. Primary collection has followed appropriate ethics guidelines.

## Results

### Data

A dataset of 3895 individuals with quality controlled, measured or imputed genotypes at 1,051,811 single nucleotide polymorphisms (SNPs) is used to test the sampling algorithms. High- (HDL-C) and low-density lipoprotein cholesterol (LDL-C, for 3822 individuals) phenotype data were available for analysis. Moreover, 20 simulated datasets were generated for four simulation configurations using the genotypes of the first chromosome (85,331 SNPs) for 2002 of the individuals and a linear model for the phenotype. The simulated data had either 30 or 100 SNPs randomly selected as causal with additive genetic effects, whose sizes were generated from a double exponential distribution. Normally distributed noise was added to the phenotypes to set the proportion of variance explained (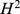) by the causal SNPs to 0.2 or 0.5. For more details on the dataset and the simulation procedure, see the previous analysis in Peltola et al. [4] and references [19], [20].

### Simulated Data

The efficiencies of the samplers were tested on the simulated datasets. The samplers are abbreviated as SS for single-step sampler, MS for multistep sampler, MS-DR for multistep sampler with delayed rejection, NK for Nott-Kohn and KSC for Kohn-Smith-Chan. Maximum move size in the multistep samplers is 20 and delayed rejection is restricted to moves with size of 10 or less. The (finite) adaptivity of SS, MS and MS-DR samplers refers to the tuning of the proposal probabilities of which variables to add or remove. Non-adaptive samplers employ discrete uniform distribution for this. All MS and MS-DR samplers use move size proposal adaptation. NK and KSC samplers were run with block sizes 1, 5 and 10. Three independent MCMC chains were run for 20,000,000 (KSC and NK) or 2,000,000 (others) iterations of the third step in the Computation and thinned by taking every 100th (KSC and NK) or every 10th (others) sample. The KSC and NK algorithms were run for ten times longer as they have cheaper iterations and showed convergence problems with shorter runs. First halves of all chains are discarded as burnin. Prior parameters are given in Text S2.

The effective sample size (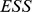) for 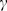 samples forms the basis of the comparisons. It estimates the number of independent samples as a ratio of the number of collected samples and the autocorrelation time (computed using Equation 4 and Geyer’s initial monotone sequence estimator [21]). We compute the geometric mean of the 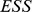 divided by the sampling time *t* (spent in step 3 of the computation) over the three chains and report relative efficiencies 
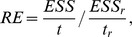
 where *r* refers to a reference.

Convergence was checked visually and by computing potential scale reduction factors [22] over all chains for model size, proportion of variance explained and 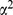 traces. These and inspection of the posterior inclusion probabilities show severe convergence and mixing problems for KSC01 and NK01 algorithms and indicate that longer runs would have been preferable on some of the dataset for other algorithms also (Table S1 and Figures S1, S2, S3, S4).

**Figure 3:**
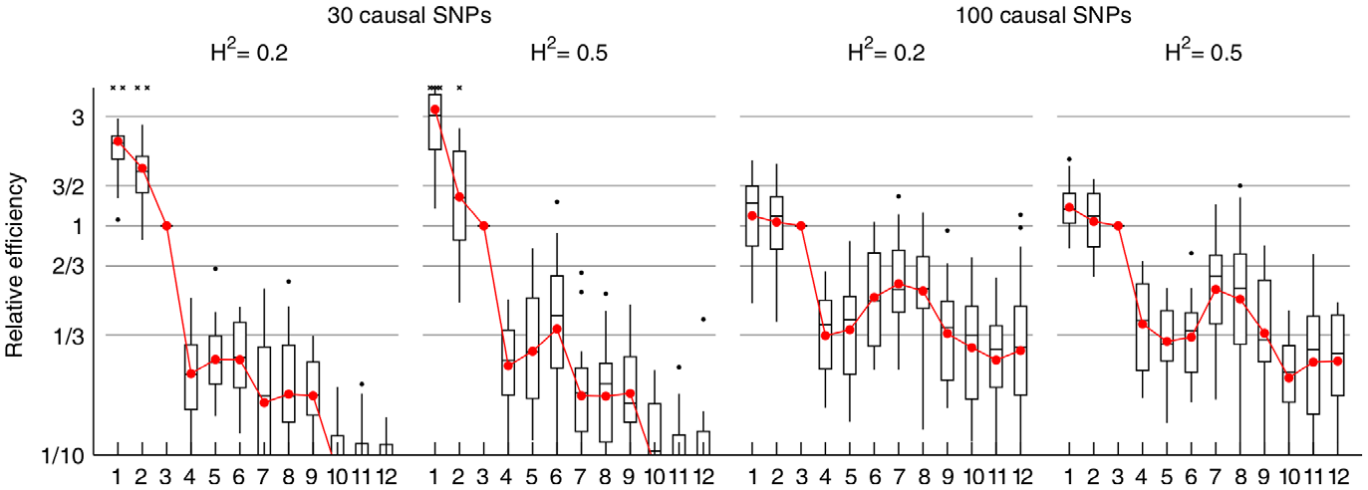
Relative efficiencies of the samplers in the simulated datasets. The boxplots show ESS/time values normalized to the third sampler, where the ESSs are computed for the 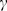 samples. Red dots show the geometric mean over the 20 datasets. Some outliers were truncated to fit into the figure and are shown with crosses. 1 = adaptive MS-DR, 2 = adaptive MS, 3 = adaptive SS, 4 = non-adaptive MS-DR, 5 = non-adaptive MS, 6 = non-adaptive SS, 7 = NK10, 8 = NK05, 9 = NK01, 10 = KSC10, 11 = KSC05, 12 = KSC01.

Figure 3 presents boxplots of the relative efficiencies, where each box represents the variation over the 20 datasets normalized to the adaptive single step sampler. The adaptive samplers have greater efficiency in all configurations of the simulations, while KSC shows the poorest performance in these datasets. Multistep moves and delayed rejection increase the efficiency especially in the simulations with 30 causal SNPs, but only in combination with the proposal distribution adaptation. The ESSs are also increased in the non-adaptive samplers with multistep moves, but less so relative to the increase in the sampling time (Table S2). KSC and NK samplers have difficulties in sampling models of different sizes (Figure S5 and Table S2).

The move size proposal distribution adaptation was validated by running the adaptive MS and MS-DR samplers with fixed move size proposal distributions for six parameter configurations (giving mean move sizes from 2 to 7) for the 20 simulated datasets with 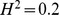 and 30 causal SNPs. The results (Table S3) indicate that the move size adaptation maximizes the realized jump distance and minimizes the first autocorrelation as intended. However, it seems that the effect of other autocorrelations on ESS is notable and, for this set of parameters and simulations, the larger the proposed move size, the larger the ESS. The differences in relative efficiencies are small (within a factor of 1.2) for the six parameter configurations.

We further note that the multistep moves and delayed rejection do not necessarily increase the efficiency of moving between different model sizes (Figure S5 and Table S2 show the relative efficiencies when the autocorrelation time is computed for model size samples). A possible explanation is that larger moves reduce the acceptance rate and a notable proportion of the moves jump between models of same size (e.g., 18% of the moves that change the model in the adaptive MS-DR sampler in the simulations with 30 causal SNPs and 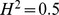 are such, while obviously none are such for the SS sampler; this comparison excludes the additional SNP switch move). Move size and rate statistics are presented in Table S4.

### HDL-C and LDL-C Data

Only the adaptive samplers proposed here were run for the HDL-C and LDL-C data as the others would be expected to perform worse with the large increase in the number of variables relative to the simulations. Twelve independent chains of length 8,000,000 iterations were run with each sampler and dataset and thinned by taking every tenth sample. Effective sample sizes and sampling times were computed as in the simulations. Here, results are presented as ESS/time rather than as relative efficiencies as there is no additional variation due to multiple datasets (HDL-C and LDL-C results are shown separately). Prior parameters are given in Text S2.

Convergence analysis did not indicate problems with the HDL-C dataset. The inferences regarding posterior inclusion probabilities and the proportion of variance explained did not change from the previous analysis [4], whereas the posterior distribution of model size is here wider reflecting the change in the effect size prior (results not shown). However, the different sampling algorithms did not converge to the same posterior distribution for the LDL-C dataset. Thus, comparisons for sampling efficiency between the samplers are not valid for the LDL-C data. On the other hand, analysis of the convergence problem is interesting.

**Figure 4:**
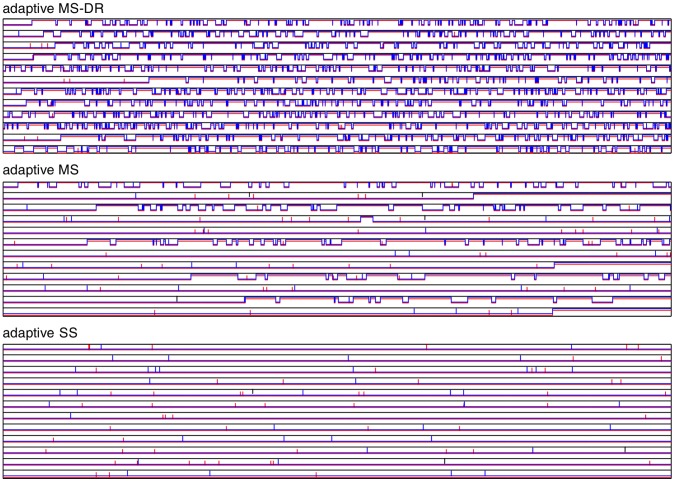
MCMC traces of the three SNPs related to convergence problems in LDL-C data. Each subplot contains traces (including burn-in period) from 12 chains, where each trace is composed of three lines (red for snp1, blue for snp2 and black for snp3), which may be in upper state (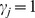) or lower state (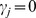). 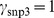 almost always, whereas 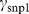 and 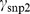 are mostly synchronized: almost always 0 for SS, often 1 for MS-DR, but changing states often and mixed for MS (some chains are like SS, some more like MS-DR but with poorer mixing).

The source of the problem is a pair of correlated (Pearson’s correlation 0.91) SNPs in the *PVRL2* gene, which have weak effects individually but a strong effect together (and preferably in combination with a third near-by SNP, which has a strong individual association). Figure 4 shows the MCMC traces for these three SNPs in all of the sampled chains. The adaptive SS sampler does not find the pair at all in these 12 chains. Most chains of the adaptive MS sampler include the pair at least at some point, but seem to mix poorly, while mixing is clearly better when delayed rejection is used. All of the samplers picked up the pair, when the dataset was reduced to contain only the SNPs in chromosome 19 (results not shown). The posterior inclusion probability for the pair is 0.79 with the MS-DR sampler. For independent evidence, a p-value of less than 0.000001 for the pair was found by computing Bayes factors using BIMBAM [23] and a million permutations of the phenotype (after adjusting for the third SNP using linear regression). Similarly computed single-SNP p-values were 0.09 and 0.15 for the two SNPs. The *PVRL2* gene is located near a region with known associations to LDL-C (e.g., the *APOE* gene) [24].

**Figure 5:**
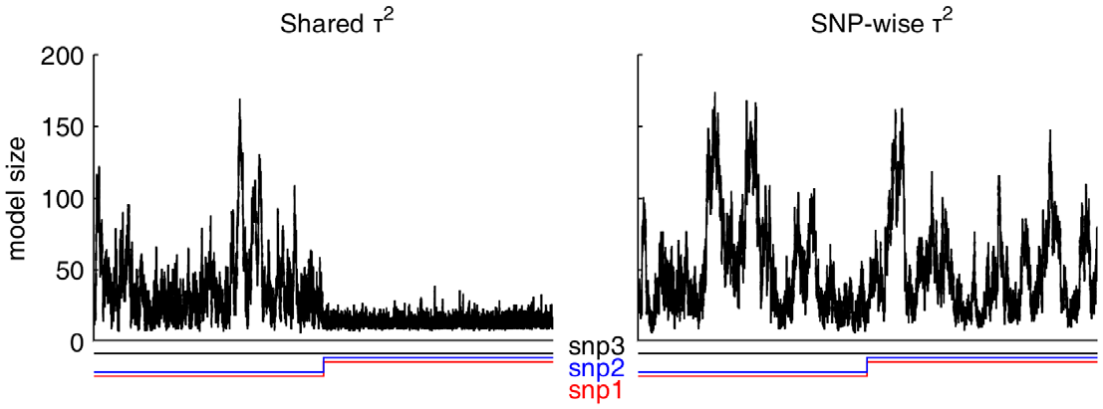
Demonstration of multimodality with shared 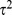
 **parameter in the LDL-C data.** The multimodality is related to the SNP pair, whose state change is shown (snp1 and snp2; 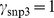 for the whole period) together with the corresponding parts of the trace of model size samples for two MCMC chains with the different priors.

The SNP pair was missed in our previous analysis [4]. This may in part have also been due to a more restricting prior for the effect sizes there (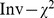 for a single variance parameter). The pair was first seen in an analysis with the noncentral-F prior for effect size variance, but with a shared 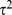 parameter. However, the prior seemed still inadequate as there was clear modal change in the shared 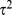 parameter to larger values on including the SNP pair in the model, which also presented as a change in the model size distribution (Figure 5). These issues spurred the change to the individual 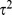 parameters and to include the second additional 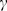 update tailored for SNP data.

**Figure 6:**
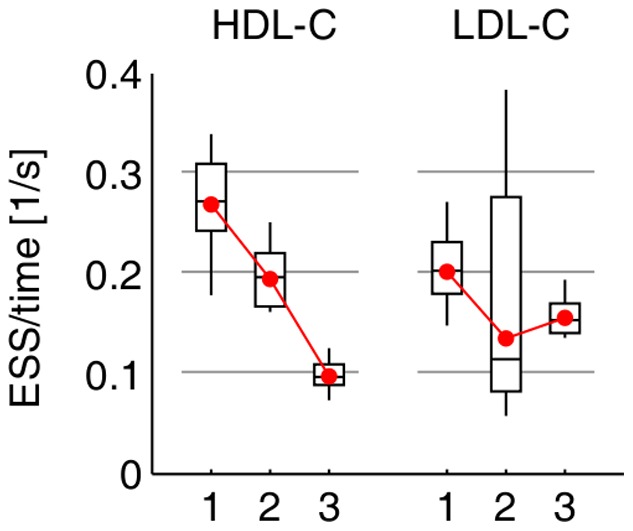
ESS/time in the HDL-C and LDL-C datasets. Boxes show the variation over the 12 independent MCMC chains for each sampler. ESSs are computed for the 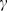 samples. Red dots show geometric means. 1 = adaptive MS-DR, 2 = adaptive MS, 3 = adaptive SS. Note that the LDL-C samplers have not converged to same posterior distribution and thus the comparison is not valid.

The ESS/time values for comparing the algorithms on sampling efficiency are shown in Figure 6 (and Table S5) for both HDL-C and LDL-C data (comparisons for the latter are invalid). Similarly to the results in simulations, the multistep moves and delayed rejection seem to increase the sampling efficiency in the HDL-C dataset. On comparing the efficiency with regard to model size samples, the trend is similar to Figure 6, but more modest (Figure S6 and Table S5).

Move size and rate statistics for the sampling algorithms are shown in Table 1. The average proposed move sizes in the multistep samplers are between 6 and 7 with the DR sampler having slightly larger values. The realized jump distance is clearly larger for the DR sampler as is the move rate, which is close to the value of the single step sampler. We note that the cutoff value for making the second stage proposal in the DR sampler (here 10) may affect the behavior of the jump distance optimization.

**Table 1:** Move size and rate statistics as averages over the MCMC chains for HDL-C and LDL-C datasets.

Dataset/Sampler	RJD	PJD	RJD/PJD	Move rate	*p*
HDL-C					
adaptive MS-DR	2.00	6.75	0.45	0.67	0.12
adaptive MS	1.15	6.25	0.33	0.33	0.14
adaptive SS	0.66	1.00	0.66	0.66	NA
LDL-C					
adaptive MS-DR	1.95	6.57	0.45	0.68	0.12
adaptive MS	1.11	6.36	0.31	0.31	0.13
adaptive SS	0.69	1.00	0.69	0.69	NA

Values are arithmetic means.

RJD: Realized jump distance (mean number of changes to 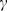 sample chain per iteration). PJD: Proposed jump distance (mean proposed number of changes per iteration). Move rate: proportion of moves with jump distance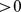 (acceptance rate for non-DR samplers). *p*: parameter of the geometric distribution for move size proposals.

## Discussion

Several aspects related to the use of the Metropolis-Hastings algorithm (MH) in Bayesian variable selection in the context of genome-wide association studies were studied here. Specifically, the focus was on the (finite) adaptation of the proposal distributions for additions and removals of variables, multistep proposals (batching of additions and removals) with move size adaptation and using a delayed rejection step in the multistep proposal. A more flexible prior formulation for the effect sizes and additional MH moves tailored to genetic data were also introduced.

The effect of the adaptation of the proposal distributions was studied on simulated datasets with 85,331 SNPs. The results suggest that the adaptation is beneficial in regard to the sampling efficiency. This is not surprising as similar ideas have been used previously in sampling from high-dimensional model spaces for variable selection [3], [4], [9], [10]. The results on simulated data and on the HDL-C dataset imply also that the multistep moves and delayed rejection (DR) are beneficial for the sampling efficiency. The DR step is similar to a block Gibbs update and it allows for oversized multistep moves, where the second stage proposal trims poor updates out. The acceptance rates for MH algorithms in large model spaces are often high and, in such cases, the DR step may also be seen to provide a short-cut relative to full Gibbs moves. The proposed algorithms were also compared to random scan versions of Nott-Kohn [9] and Kohn-Smith-Chan [18] samplers. These seemed to have problems especially in moving along the model size distribution and showed worse performance than the proposed finitely adaptive algorithms in all configurations of the simulated data.

The expected jump distance optimization [13], used here for adapting the move size proposals, provides an alternative to relying on the knowledge of an optimal acceptance rate. However, it has two caveats: the optimization does not account for the increase in computational effort for larger move sizes (there is no such problem with a Gaussian proposal distribution) and, in our limited experiments, minimizing the first autocorrelation did not lead to a minimum of the autocorrelation time. The acceptance rates of the multistep moves (without DR) fell in 0.30–0.42 for all experiments in this work, which corresponds well with the empirical optimal range of 0.25–0.40 found by Lamnisos et al. [25] in the case of variable selection for probit regression.

Problems in the mixing of the samplers were found in the LDL-C data. This was identified being related to a pair of SNPs, which are required to be together in the model to have notable contribution. The interpretation of the SNP pair is unclear to us (e.g., haplotype tag or false positive), but it is plausible that such combinations could be found in other datasets also and that they are probably missed in single-SNP analyses. Multistep moves may help in finding such SNP pairs, but it is still improbable that one move would happen to propose the correct pair amongst all possible. We introduced a specific MH move to alleviate the problem of finding such local SNP combinations. Together with the delayed rejection, which allows for some misspecification of move size, this seemed to improve the mixing for the SNP pair markedly.

Moreover, the prior distribution of the effect sizes was changed to have more probability mass near the axes for the regression coefficients (through having SNP specific 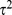 parameters), which may be more appropriate in cases where there are large differences in the effect sizes of associated variables. This seems desirable in genome-wide association analysis. Having a shared 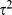 parameter led to multimodal posterior distributions for 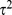 and model size in the LDL-C data. Such behavior was not observed with the more flexible prior. However, the issue highlights the potential sensitivity of the model size posterior to the prior specification, which has been long acknowledged in the literature on Bayesian varying dimensional models (e.g., [26]).

We acknowledge that comparisons for sampling efficiency may be sensitive to the implementation, sampling parameters and the computer environment, where the experiments are run. To this end, all experiments here were run on a cluster computer, where the nodes have almost identical configurations (most importantly, the same CPU model and software libraries for linear algebra; for HDL-C, and similarly for LDL-C, a single node was used to run all experiments) and the same sampling parameters were used for all algorithms (where applicable). Moreover, the third step in the Gibbs scheme, the variable inclusion update, was timed separately and was used to compute the efficiencies. Thus, the time spent in the other steps, which may account for a significant portion of the total time (especially the Rao-Blackwellization), was excluded. All of the algorithms were implemented by the first author and most of the source code is shared between them. A set of unit tests (including checks for likelihood computations and sampling on small test data, among others) was used to increase confidence in the correctness of the implementation and is available with the source code.

The results may also be expected to vary with the specifics of the data (e.g., scale, number of significant associations, effect size distribution and correlation structure) as seen to some extent between the different simulation configurations. Our experiments were specifically in the context of genome-wide association analysis, but many of the ideas are applicable to other types of high-dimensional data. However, the sampling algorithms used here may need to be combined with other means of tackling potential multimodality for general use.

## Supporting Information

Figure S1 **Model size posterior distributions in the simulated data (three estimated densities per method).**
(TIF)

Figure S2 **Model size posterior distributions in the simulated data (three estimated densities per method).**
(TIF)

Figure S3 **Model size posterior distributions in the simulated data (three estimated densities per method).**
(TIF)

Figure S4 **Model size posterior distributions in the simulated data (three estimated densities per method).**
(TIF)

Figure S5 **Boxplot of the relative efficiencies (ESS/time normalized to third sampler) of the samplers in the simulation datasets computed for the model size samples.** Red dots show the geometric mean over the 20 datasets. 1 = adaptive MS-DR, 2 = adaptive MS, 3 = adaptive SS, 4 = non-adaptive MS-DR, 5 = non-adaptive MS, 6 = non-adaptive SS, 7 = NK10, 8 = NK05, 9 = NK01, 10 = KSC10, 11 = KSC05, 12 = KSC01.
(TIF)

Figure S6 **ESS/time boxplot, where ESS is computed based on the autocorrelation of model size samples for the HDL-C and LDL-C datasets.** 1 = adaptive MS-DR, 2 = adaptive MS, 3 = adaptive SS.
(TIF)

Table S1 **Posterior inclusion probability consistency for the simulated datasets.**
(PDF)

Table S2 **Sampling time, ESS, ESS/time and relative efficiency for the simulated datasets.**
(PDF)

Table S3 **Efficiency and move size statistics for fixed move size proposal distribution sampling experiments.**
(PDF)

Table S4 **Move size and rate statistics as averages over the 20 simulation datasets.**
(PDF)

Table S5 **Sampling time, ESS, ESS/time and relative efficiency for the LDL-C and HDL-C datasets.**
(PDF)

Text S1 **Supplementary methods.**
(PDF)

Text S2 **Prior parameters for the simulation, HDL-C and LDL-C models.**
(PDF)

## References

[1] Hindorff LA, MacArthur J, Wise A, Junkins HA, Hall P, et al. (2012) A catalog of published genome-wide association studies. Available: www.genome.gov/gwastudies. Accessed 2012 Mar 28.

[2] Hoggart CJ, Whittaker JC, De Iorio M, Balding DJ (2008) Simultaneous analysis of all SNPs in genome-wide and re-sequencing association studies. PLoS Genet 4: e1000130. 18654633 10.1371/journal.pgen.1000130 PMC2464715

[3] Guan Y, Stephens M (2011) Bayesian variable selection regression for genome-wide association studies, and other large-scale problems. Ann Appl Stat 5: 1780–1815.

[4] Peltola T, Marttinen P, Jula A, Salomaa V, Perola M, et al (2012) Bayesian variable selection in searching for additive and dominant effects in genome-wide data. PLoS ONE 7: e29115. 22235263 10.1371/journal.pone.0029115 PMC3250410

[5] Metropolis N, Rosenbluth AW, Rosenbluth MN, Teller AH, Teller E (1953) Equation of state calculations by fast computing machines. J Chem Phys 21: 1087.

[6] Hastings WK (1970) Monte Carlo sampling methods using Markov chains and their applications. Biometrika 57: 97–109.

[7] Mira A (2001) On Metropolis-Hastings algorithms with delayed rejection. Metron 59: 231–241.

[8] Green PJ, Mira A (2001) Delayed rejection in reversible jump Metropolis-Hastings. Biometrika 88: 1035–1053.

[9] Nott DJ, Kohn R (2005) Adaptive sampling for bayesian variable selection. Biometrika 92: 747–763.

[10] Clyde MA, Ghosh J, Littman ML (2011) Bayesian adaptive sampling for variable selection and model averaging. J Comput Graph Stat 20: 80–101.

[11] Storvik G (2011) On the exibility of Metropolis-Hastings acceptance probabilities in auxiliary variable proposal generation. Scand J Stat 38: 342–358.

[12] Lamnisos D, Griffin JE, Steel MF (2011) Adaptive Monte Carlo for Bayesian variable selection in regression models. Technical report, CRiSM Working Paper 09–41, revised version.

[13] Pasarica C, Gelman A (2010) Adaptively scaling the Metropolis algorithm using expected squared jumped distance. Stat Sinica 20: 343–364.

[14] Bottolo L, Richardson S (2010) Evolutionary stochastic search for Bayesian model exploration. Bayesian Anal 5: 583–618.

[15] Wilson MA, Iversen ES, Clyde MA, Schmidler SC, Schildkraut JM (2010) Bayesian model search and multilevel inference for SNP association studies. Ann Appl Stat 4: 1342–1364. 21179394 10.1214/09-aoas322 PMC3004292

[16] Gelman A (2006) Prior distributions for variance parameters in hierarchical models (comment on article by Browne and Draper). Bayesian Anal 1: 515–534.

[17] Peskun P (1973) Optimum Monte-Carlo sampling using Markov chains. Biometrika 60: 607.

[18] Kohn R, Smith M, Chan D (2001) Nonparametric regression using linear combinations of basis functions. Stat Comput 11: 313–322.

[19] Perttilä J, Merikanto K, Naukkarinen J, Surakka I, Martin NW, et al (2009) OSBPL10, a novel candidate gene for high triglyceride trait in dyslipidemic Finnish subjects, regulates cellular lipid metabolism. J Mol Med 87: 825–835. 19554302 10.1007/s00109-009-0490-z PMC2707950

[20] Vartiainen E, Laatikainen T, Peltonen M, Juolevi A, Männistö S, et al (2010) Thirty-five-year trends in cardiovascular risk factors in Finland. Int J Epidemiol 39: 504–518. 19959603 10.1093/ije/dyp330

[21] Geyer CJ (1992) Practical Markov chain Monte Carlo. Stat Sci 7: 473–511.

[22] Gelman A, Carlin JB, Stern HS, Rubin DB (2004) Bayesian data analysis. Chapman & Hall/CRC, pp294–299.

[23] Guan Y, Stephens M (2008) Practical issues in imputation-based association mapping. PLoS Genet 4: e1000279. 19057666 10.1371/journal.pgen.1000279 PMC2585794

[24] Teslovich TM, Musunuru K, Smith AV, Edmondson AC, Stylianou IM, et al (2010) Biological, clinical and population relevance of 95 loci for blood lipids. Nature 466: 707–713. 20686565 10.1038/nature09270 PMC3039276

[25] Lamnisos D, Griffin JE, Steel MF (2009) Transdimensional sampling algorithms for Bayesian variable selection in classification problems with many more variables than observations. J Comput Graph Stat 18: 592–612.

[26] Richardson S, Green PJ (1997) On Bayesian analysis of mixtures with an unknown number of components. J Roy Stat Soc B 59: 731–792.

