# Supplementary figures and images for: Finite Adaptation and Multistep Moves in the Metropolis-Hastings Algorithm for Variable Selection in Genome-Wide Association Analysis
Source: PLoS One. 2012 Nov 15;7(11):e49445. doi: 10.1371/journal.pone.0049445 (PMC3499564; doi:10.1371/journal.pone.0049445)

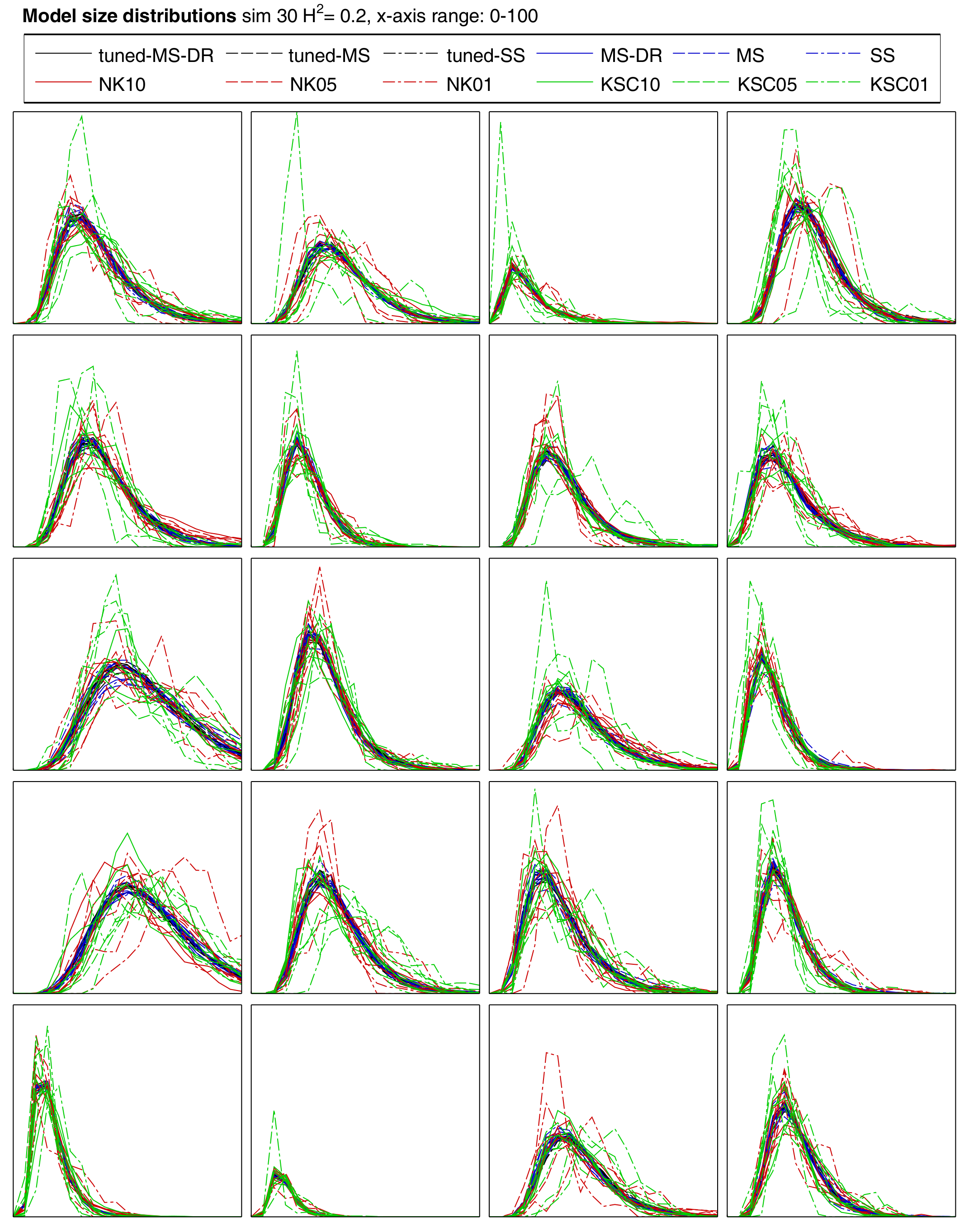

Supplement: Figure S1 — Model size posterior distributions in the simulated data (three estimated densities per method). (TIF) [file pone.0049445.s001.tif]

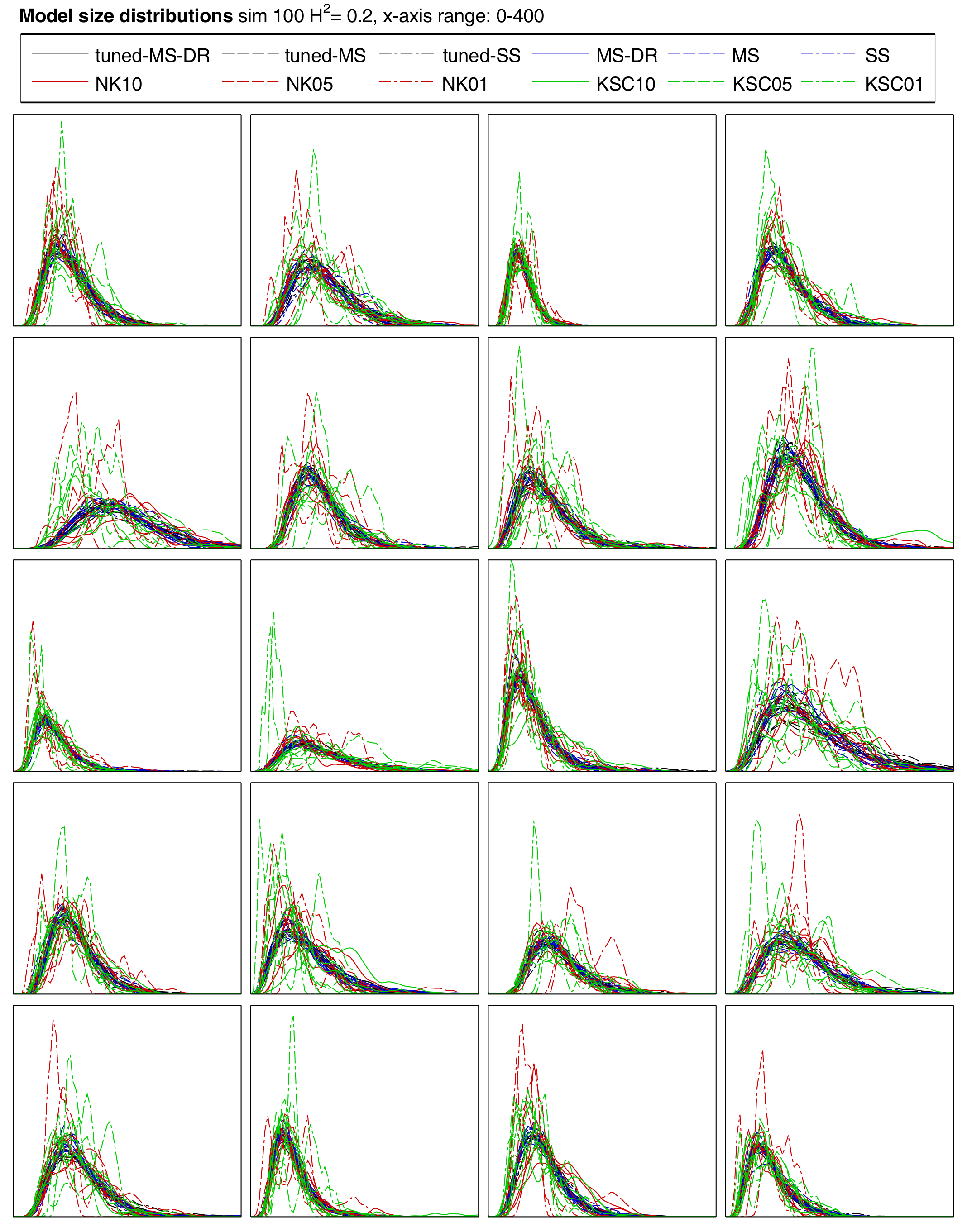

Supplement: Figure S2 — Model size posterior distributions in the simulated data (three estimated densities per method). (TIF) [file pone.0049445.s002.tif]

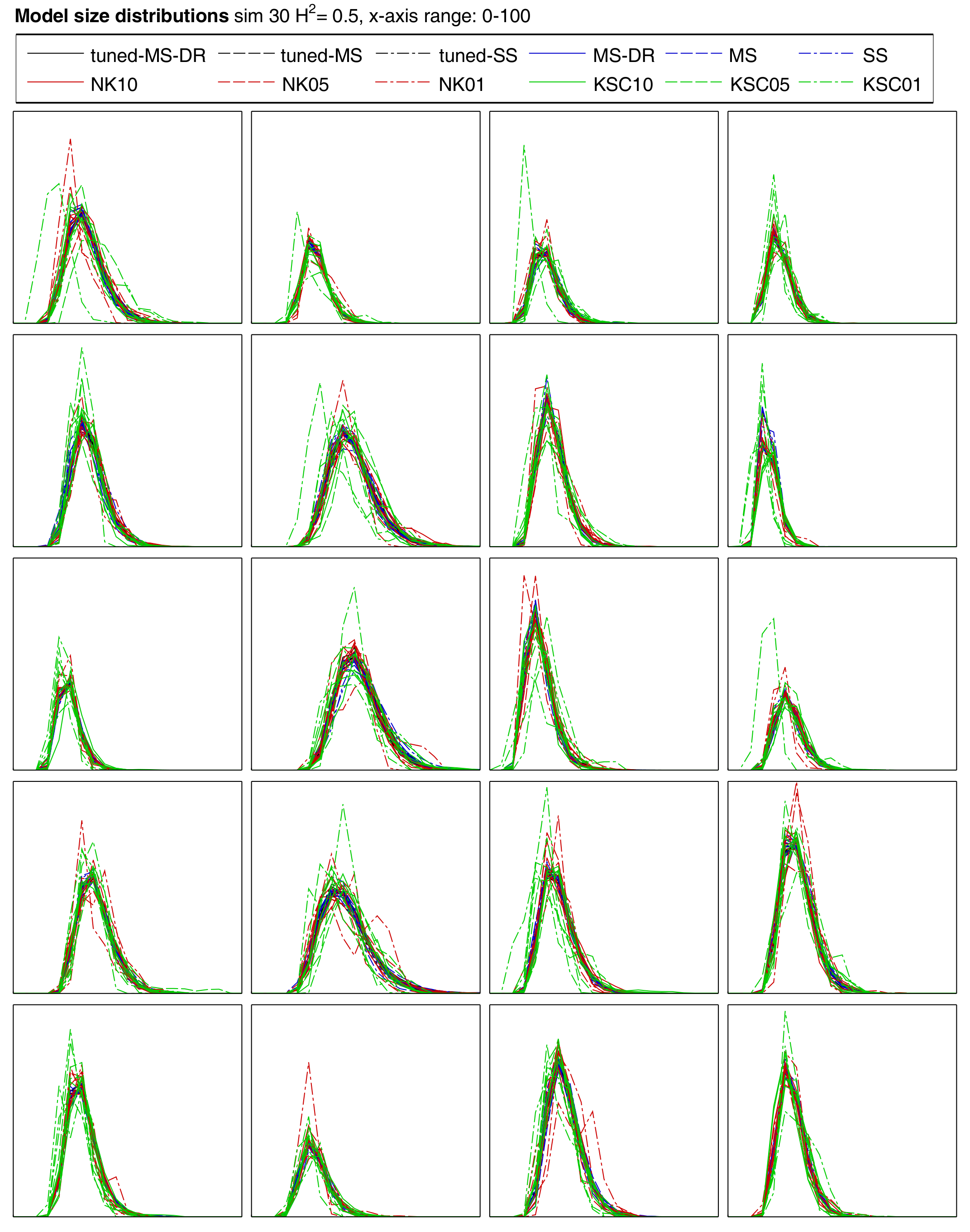

Supplement: Figure S3 — Model size posterior distributions in the simulated data (three estimated densities per method). (TIF) [file pone.0049445.s003.tif]

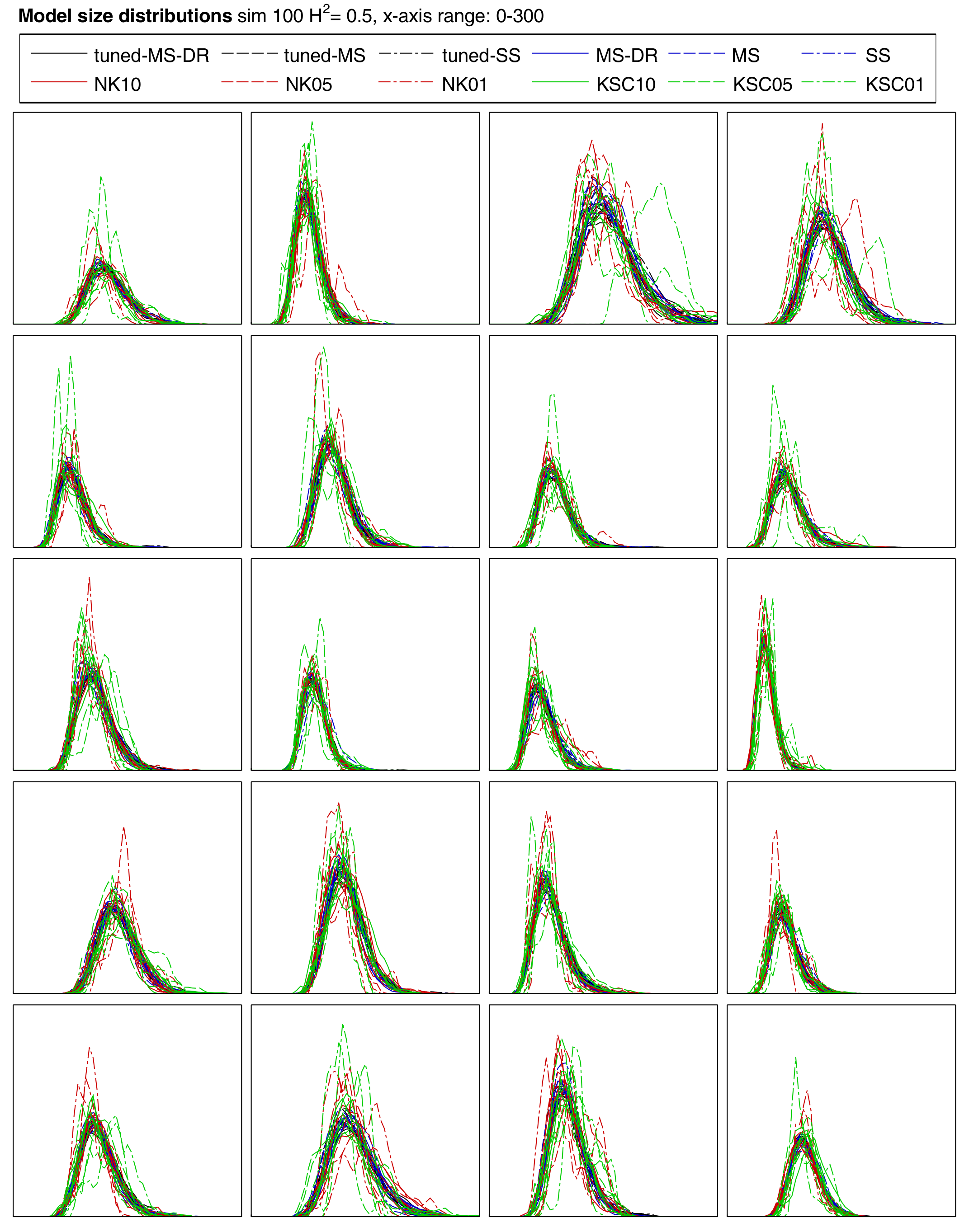

Supplement: Figure S4 — Model size posterior distributions in the simulated data (three estimated densities per method). (TIF) [file pone.0049445.s004.tif]

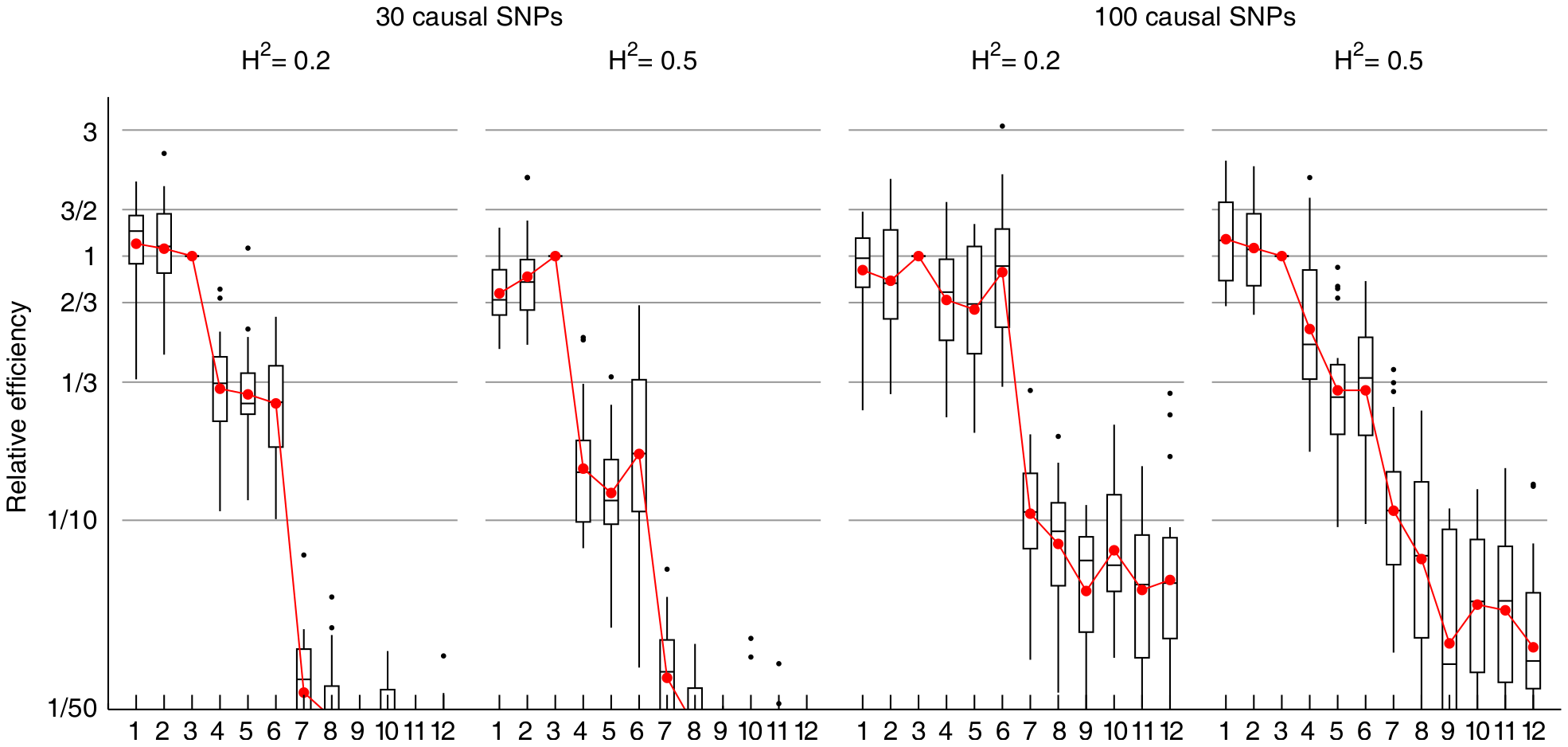

Supplement: Figure S5 — Boxplot of the relative efficiencies (ESS/time normalized to third sampler) of the samplers in the simulation datasets computed for the model size samples. Red dots show the geometric mean over the 20 datasets. 1 = adaptive MS-DR, 2 = adaptive MS, 3 = adaptive SS, 4 = non-adaptive MS-DR, 5 = non-adaptive MS, 6 = non-adaptive SS, 7 = NK10, 8 = NK05, 9 = NK01, 10 = KSC10, 11 = KSC05, 12 = KSC01. (TIF) [file pone.0049445.s005.tif]

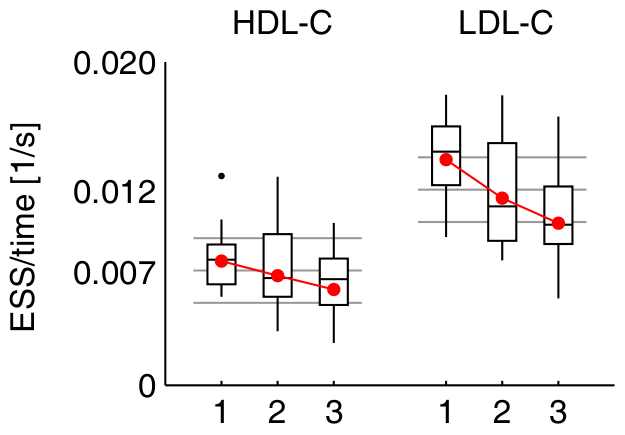

Supplement: Figure S6 — ESS/time boxplot, where ESS is computed based on the autocorrelation of model size samples for the HDL-C and LDL-C datasets. 1 = adaptive MS-DR, 2 = adaptive MS, 3 = adaptive SS. (TIF) [file pone.0049445.s006.tif]
