# Supplementary material for: Finite Adaptation and Multistep Moves in the Metropolis-Hastings Algorithm for Variable Selection in Genome-Wide Association Analysis
Source: PLoS One. 2012 Nov 15;7(11):e49445. doi: 10.1371/journal.pone.0049445 (PMC3499564; doi:10.1371/journal.pone.0049445)
Supplement: Table S1 — Posterior inclusion probability consistency for the simulated datasets. (PDF) [file pone.0049445.s007.pdf]

Finite Adaptation and Multistep Moves in the Metropolis-Hastings Algorithm for Variable Selection in Genome-Wide Association Analysis

Supplementary Table S1

Tomi Peltola, Pekka Marttinen, and Aki Vehtari

Posterior inclusion probability consistency

	intra-correlation			inter-correlation		
Setting / Sampler	median	IQR	range	median	IQR	range
30 causal SNPs, $H^2 = 0.2$						
adaptive MS-DR	1.00	1.00–1.00	0.99–1.00	1.00	1.00–1.00	1.00–1.00
adaptive MS	1.00	1.00–1.00	0.99–1.00	1.00	1.00–1.00	0.99–1.00
adaptive SS	1.00	0.99–1.00	0.98–1.00	1.00	1.00–1.00	0.99–1.00
non-adaptive MS-DR	0.99	0.99–1.00	0.98–1.00	1.00	0.99–1.00	0.99–1.00
non-adaptive MS	0.99	0.98–0.99	0.93–1.00	0.99	0.99–1.00	0.97–1.00
non-adaptive SS	0.99	0.98–0.99	0.93–1.00	0.99	0.99–1.00	0.96–1.00
NK10	0.99	0.99–1.00	0.93–1.00	1.00	0.99–1.00	0.95–1.00
NK05	1.00	0.99–1.00	0.92–1.00	1.00	0.99–1.00	0.96–1.00
NK01	0.99	0.97–1.00	0.90–1.00	1.00	0.99–1.00	0.93–1.00
KSC10	0.99	0.98–1.00	0.94–1.00	1.00	0.99–1.00	0.97–1.00
KSC05	0.99	0.97–0.99	0.90–1.00	1.00	0.98–1.00	0.96–1.00
KSC01	0.97	0.94–0.98	0.81–1.00	0.98	0.97–0.99	0.84–1.00
100 causal SNPs, $H^2 = 0.2$						
adaptive MS-DR	1.00	0.99–1.00	0.99–1.00	1.00	1.00–1.00	0.99–1.00
adaptive MS	1.00	0.99–1.00	0.99–1.00	1.00	1.00–1.00	1.00–1.00
adaptive SS	0.99	0.98–0.99	0.93–1.00	0.99	0.99–1.00	0.96–1.00
non-adaptive MS-DR	0.98	0.97–0.99	0.93–0.99	0.99	0.99–0.99	0.97–1.00
non-adaptive MS	0.98	0.97–0.99	0.95–0.99	0.99	0.99–0.99	0.97–1.00
non-adaptive SS	0.97	0.97–0.98	0.93–0.99	0.99	0.98–0.99	0.95–1.00
NK10	1.00	0.99–1.00	0.96–1.00	1.00	1.00–1.00	0.98–1.00
NK05	0.99	0.99–1.00	0.98–1.00	1.00	1.00–1.00	0.98–1.00
NK01	0.99	0.98–0.99	0.86–1.00	0.99	0.99–1.00	0.95–1.00
KSC10	0.99	0.99–0.99	0.95–1.00	1.00	0.99–1.00	0.97–1.00
KSC05	0.99	0.98–0.99	0.96–1.00	0.99	0.99–1.00	0.98–1.00
KSC01	0.98	0.97–0.98	0.94–0.99	0.99	0.99–0.99	0.97–1.00

Setting / Sampler	intra-correlation median	IQR	range	inter-correlation median	IQR	range
30 causal SNPs, $H^2 = 0.5$						
adaptive MS-DR	1.00	1.00–1.00	0.98–1.00	1.00	1.00–1.00	0.99–1.00
adaptive MS	1.00	1.00–1.00	0.91–1.00	1.00	1.00–1.00	0.94–1.00
adaptive SS	1.00	0.99–1.00	0.93–1.00	1.00	0.99–1.00	0.97–1.00
non-adaptive MS-DR	0.99	0.99–1.00	0.92–1.00	1.00	0.99–1.00	0.97–1.00
non-adaptive MS	0.99	0.98–0.99	0.94–1.00	0.99	0.99–1.00	0.92–1.00
non-adaptive SS	0.99	0.98–1.00	0.89–1.00	0.99	0.99–1.00	0.95–1.00
NK10	1.00	0.98–1.00	0.87–1.00	1.00	0.99–1.00	0.93–1.00
NK05	0.99	0.99–1.00	0.85–1.00	1.00	0.99–1.00	0.94–1.00
NK01	0.99	0.97–1.00	0.83–1.00	0.99	0.99–1.00	0.94–1.00
KSC10	0.99	0.97–0.99	0.86–1.00	0.99	0.99–1.00	0.93–1.00
KSC05	0.99	0.97–0.99	0.88–1.00	0.99	0.99–1.00	0.91–1.00
KSC01	0.90	0.76–0.95	0.34–1.00	0.96	0.86–0.99	0.43–1.00
100 causal SNPs, $H^2 = 0.5$						
adaptive MS-DR	0.99	0.99–1.00	0.99–1.00	1.00	1.00–1.00	0.99–1.00
adaptive MS	0.99	0.99–0.99	0.94–1.00	0.99	0.99–1.00	0.95–1.00
adaptive SS	0.99	0.98–0.99	0.96–0.99	0.99	0.99–0.99	0.97–1.00
non-adaptive MS-DR	0.98	0.97–0.98	0.94–0.99	0.99	0.99–0.99	0.98–1.00
non-adaptive MS	0.97	0.96–0.98	0.87–0.99	0.98	0.98–0.99	0.93–0.99
non-adaptive SS	0.97	0.96–0.98	0.94–0.99	0.98	0.98–0.99	0.96–0.99
NK10	0.99	0.99–1.00	0.93–1.00	1.00	0.99–1.00	0.96–1.00
NK05	0.99	0.98–0.99	0.96–1.00	1.00	0.99–1.00	0.97–1.00
NK01	0.98	0.97–0.99	0.94–1.00	0.99	0.99–1.00	0.96–1.00
KSC10	0.99	0.98–0.99	0.93–1.00	0.99	0.99–1.00	0.97–1.00
KSC05	0.98	0.98–0.99	0.94–0.99	0.99	0.99–0.99	0.97–1.00
KSC01	0.98	0.96–0.98	0.93–0.99	0.99	0.98–0.99	0.94–1.00

Intra-correlation: Correlations between the three chains (chain 1 vs chain 2, 1 vs 3, 2 vs 3) pooled for the 20 datasets in each simulation configuration and the statistics computed from the 60 (20 x 3) values for each sampler.

Inter-correlation: Correlations of each chain against the mean posterior inclusion probabilities over all chains and samplers (chain 1 vs mean, 2 vs mean, 3 vs mean) for the 20 datasets pooled and the statistics computed from the 60 (20 x 3) values for each sampler.

Statistics: median, inter-quartile range (IQR) and range (minimum and maximum values).
