# Supplementary material for: Finite Adaptation and Multistep Moves in the Metropolis-Hastings Algorithm for Variable Selection in Genome-Wide Association Analysis
Source: PLoS One. 2012 Nov 15;7(11):e49445. doi: 10.1371/journal.pone.0049445 (PMC3499564; doi:10.1371/journal.pone.0049445)
Supplement: Table S2 — Sampling time, ESS, ESS/time and relative efficiency for the simulated datasets. (PDF) [file pone.0049445.s008.pdf]

Finite Adaptation and Multistep Moves in the Metropolis-Hastings Algorithm for Variable Selection in Genome-Wide Association Analysis

Supplementary Table S2

Tomi Peltola, Pekka Marttinen, and Aki Vehtari

Sampling time, ESS, ESS/time and relative efficiency for the simulated datasets.

Geometric mean values of sampling time (seconds), ESS, ESS/time and relative efficiency for the simulated datasets. ESSs are based on the autocorrelation of γ and model size samples.

		γ			model size		
Setting / Sampler	time	ESS	ESS/time	RE	ESS	ESS/time	RE
30 causal SNPs, $H^2 = 0.2$							
adaptive MS-DR	1079	5430	5.03	2.3	735	0.68	1.1
adaptive MS	783	3010	3.84	1.8	511	0.65	1.1
adaptive SS	586	1262	2.15	1.0	358	0.61	1.0
non-adaptive MS-DR	1418	690	0.49	0.2	273	0.19	0.3
non-adaptive MS	851	478	0.56	0.3	156	0.18	0.3
non-adaptive SS	723	405	0.56	0.3	122	0.17	0.3
NK10	3422	1246	0.36	0.2	47	0.01	0.0
NK05	2736	1085	0.40	0.2	30	0.01	0.0
NK01	1508	588	0.39	0.2	10	0.01	0.0
KSC10	3083	557	0.18	0.1	29	0.01	0.0
KSC05	2872	453	0.16	0.1	17	0.01	0.0
KSC01	1759	308	0.17	0.1	9	0.01	0.0
30 causal SNPs, $H^2 = 0.5$							
adaptive MS-DR	967	3211	3.32	3.2	938	0.97	0.7
adaptive MS	626	863	1.38	1.3	702	1.12	0.8
adaptive SS	488	503	1.03	1.0	654	1.34	1.0
non-adaptive MS-DR	1177	297	0.25	0.2	248	0.21	0.2
non-adaptive MS	684	200	0.29	0.3	116	0.17	0.1
non-adaptive SS	552	202	0.37	0.4	132	0.24	0.2
NK10	3166	592	0.19	0.2	108	0.03	0.0
NK05	2598	483	0.19	0.2	57	0.02	0.0
NK01	1637	314	0.19	0.2	19	0.01	0.0
KSC10	2635	246	0.09	0.1	40	0.02	0.0
KSC05	2635	214	0.08	0.1	24	0.01	0.0
KSC01	1608	136	0.08	0.1	11	0.01	0.0

Setting / Sampler	time	γ ESS	γ ESS/time	γ RE	model size ESS	model size ESS/time	model size RE
100 causal SNPs, $H^2 = 0.2$							
adaptive MS-DR	7147	2923	0.41	1.1	135	0.02	0.9
adaptive MS	6162	2366	0.38	1.0	106	0.02	0.8
adaptive SS	2470	914	0.37	1.0	53	0.02	1.0
non-adaptive MS-DR	6455	792	0.12	0.3	94	0.01	0.7
non-adaptive MS	4675	608	0.13	0.4	63	0.01	0.6
non-adaptive SS	2223	401	0.18	0.5	41	0.02	0.9
NK10	8890	1836	0.21	0.6	20	0.00	0.1
NK05	7530	1447	0.19	0.5	13	0.00	0.1
NK01	6198	777	0.13	0.3	7	0.00	0.1
KSC10	10139	1103	0.11	0.3	17	0.00	0.1
KSC05	9363	899	0.10	0.3	11	0.00	0.1
KSC01	4941	522	0.11	0.3	6	0.00	0.1
100 causal SNPs, $H^2 = 0.5$							
adaptive MS-DR	4747	1031	0.22	1.2	312	0.07	1.2
adaptive MS	2935	553	0.19	1.0	178	0.06	1.1
adaptive SS	2326	420	0.18	1.0	132	0.06	1.0
non-adaptive MS-DR	4156	279	0.07	0.4	125	0.03	0.5
non-adaptive MS	3480	196	0.06	0.3	61	0.02	0.3
non-adaptive SS	3122	184	0.06	0.3	55	0.02	0.3
NK10	7964	758	0.10	0.5	49	0.01	0.1
NK05	6965	601	0.09	0.5	28	0.00	0.1
NK01	5493	337	0.06	0.3	11	0.00	0.0
KSC10	10125	396	0.04	0.2	28	0.00	0.0
KSC05	7182	330	0.05	0.3	19	0.00	0.0
KSC01	4962	230	0.05	0.3	9	0.00	0.0
