# Supplementary material for: Finite Adaptation and Multistep Moves in the Metropolis-Hastings Algorithm for Variable Selection in Genome-Wide Association Analysis
Source: PLoS One. 2012 Nov 15;7(11):e49445. doi: 10.1371/journal.pone.0049445 (PMC3499564; doi:10.1371/journal.pone.0049445)
Supplement: Table S3 — Efficiency and move size statistics for fixed move size proposal distribution sampling experiments. (PDF) [file pone.0049445.s009.pdf]

Finite Adaptation and Multistep Moves in the Metropolis-Hastings Algorithm for Variable Selection in Genome-Wide Association Analysis

Supplementary Table S3

Tomi Peltola, Pekka Marttinen, and Aki Vehtari

Efficiency and move size statistics for fixed move size proposal distribution sampling experiments

Geometric mean values of sampling time (seconds), ESS, ESS/time, relative efficiency (normalized to the sampler with mean move size of 2) and arithmetic mean values of move size and rate statistics for the simulated datasets with $H^2 = 0.2$ and 30 causal SNPs with fixed move size proposal distribution samplers. ESSs are based on the autocorrelation of $\boldsymbol{\gamma}$ samples. RJD = Realized jump distance. PJD = Proposed jump distance. Move rate = proportion of moves with jump distance > 0 (acceptance rate for non-DR samplers). p is the (fixed) parameter of the move size proposal distribution.

Sampler	Time	ρ_1	ESS	ESS/time	RE	RJD	PJD	RJD/PJD	Move rate	p
adaptive MS20-DR10										
mean PJD 2	521	0.840	2608	5.00	1.0	1.16	2.00	0.62	0.73	0.50
mean PJD 3	635	0.803	3718	5.86	1.2	1.52	3.00	0.58	0.76	0.33
mean PJD 4	781	0.781	4528	5.80	1.2	1.73	4.01	0.54	0.76	0.25
mean PJD 5	888	0.771	5041	5.68	1.1	1.84	5.00	0.50	0.74	0.19
mean PJD 6	928	0.766	5264	5.67	1.1	1.89	6.00	0.46	0.70	0.14
mean PJD 7	1001	0.767	5492	5.48	1.1	1.88	7.00	0.42	0.66	0.11
adaptive MS20										
mean PJD 2	480	0.870	2157	4.50	1.0	0.89	2.00	0.54	0.54	0.50
mean PJD 3	581	0.861	2629	4.53	1.0	0.96	3.00	0.46	0.46	0.33
mean PJD 4	637	0.857	2987	4.69	1.0	0.99	4.01	0.40	0.40	0.25
mean PJD 5	687	0.859	3126	4.55	1.0	0.98	5.00	0.35	0.35	0.19
mean PJD 6	685	0.860	3267	4.77	1.1	0.97	6.00	0.31	0.31	0.14
mean PJD 7	746	0.861	3310	4.44	1.0	0.94	7.00	0.27	0.27	0.11
