# Supplementary material for: Finite Adaptation and Multistep Moves in the Metropolis-Hastings Algorithm for Variable Selection in Genome-Wide Association Analysis
Source: PLoS One. 2012 Nov 15;7(11):e49445. doi: 10.1371/journal.pone.0049445 (PMC3499564; doi:10.1371/journal.pone.0049445)
Supplement: Table S4 — Move size and rate statistics as averages over the 20 simulation datasets. (PDF) [file pone.0049445.s010.pdf]

Finite Adaptation and Multistep Moves in the Metropolis-Hastings Algorithm for Variable Selection in Genome-Wide Association Analysis

Supplementary Table S4

Tomi Peltola, Pekka Marttinen, and Aki Vehtari

Move size and rate statistics as averages over the 20 simulation datasets

Move size and rate statistics as averages over the 20 simulation datasets. Values are arithmetic means. RJD = Realized jump distance. PJD = Proposed jump distance. Move rate = proportion of moves with jump distance > 0 (acceptance rate for non-DR samplers). p is the parameter of the move size proposal distribution.

Setting / Sampler	RJD	PJD	RJD/PJD	Move rate	p
30 causal SNPs, $H^2 = 0.2$					
adaptive MS-DR	1.90	6.32	0.45	0.68	0.13
adaptive MS	1.02	4.36	0.39	0.39	0.24
adaptive SS	0.67	1.00	0.67	0.67	NA
non-adaptive MS-DR	1.26	5.86	0.32	0.60	0.15
non-adaptive MS	0.53	2.14	0.34	0.34	0.48
non-adaptive SS	0.47	1.00	0.47	0.47	NA
30 causal SNPs, $H^2 = 0.5$					
adaptive MS-DR	1.32	5.80	0.34	0.63	0.15
adaptive MS	0.57	1.73	0.42	0.42	0.61
adaptive SS	0.53	1.00	0.53	0.53	NA
non-adaptive MS-DR	0.82	5.86	0.21	0.48	0.15
non-adaptive MS	0.30	1.10	0.29	0.29	0.91
non-adaptive SS	0.30	1.00	0.30	0.30	NA
100 causal SNPs, $H^2 = 0.2$					
adaptive MS-DR	2.71	8.79	0.46	0.62	0.05
adaptive MS	1.97	9.73	0.31	0.31	0.02
adaptive SS	0.76	1.00	0.76	0.76	NA
non-adaptive MS-DR	1.90	6.68	0.43	0.66	0.12
non-adaptive MS	1.04	5.67	0.32	0.32	0.17
non-adaptive SS	0.63	1.00	0.63	0.63	NA
100 causal SNPs, $H^2 = 0.5$					
adaptive MS-DR	1.55	5.80	0.40	0.67	0.15
adaptive MS	0.72	2.66	0.40	0.40	0.39
adaptive SS	0.60	1.00	0.60	0.60	NA
non-adaptive MS-DR	1.20	5.86	0.31	0.59	0.15
non-adaptive MS	0.49	1.90	0.34	0.34	0.54
non-adaptive SS	0.45	1.00	0.45	0.45	NA
