# Supplementary material for: Finite Adaptation and Multistep Moves in the Metropolis-Hastings Algorithm for Variable Selection in Genome-Wide Association Analysis
Source: PLoS One. 2012 Nov 15;7(11):e49445. doi: 10.1371/journal.pone.0049445 (PMC3499564; doi:10.1371/journal.pone.0049445)
Supplement: Table S5 — Sampling time, ESS, ESS/time and relative efficiency for the LDL-C and HDL-C datasets. (PDF) [file pone.0049445.s011.pdf]

Finite Adaptation and Multistep Moves in the Metropolis-Hastings Algorithm for Variable Selection in Genome-Wide Association Analysis

Supplementary Table S5

Tomi Peltola, Pekka Marttinen, and Aki Vehtari

Sampling time, ESS, ESS/time and relative efficiency for the LDL-C and HDL-C datasets

Geometric mean values of sampling time (seconds), ESS, ESS/time and relative efficiency for the simulated datasets. ESSs are based on the autocorrelation of γ and model size samples.

		γ			model size		
Setting / Sampler	Time	ESS	ESS/time	RE	ESS	ESS/time	RE
HDL-C							
adaptive MS-DR	45205	12080	0.27	2.8	343	0.01	1.3
adaptive MS	42753	8240	0.19	2.0	286	0.01	1.1
adaptive SS	35005	3346	0.10	1.0	204	0.01	1.0
LDL-C							
adaptive MS-DR	41071	8216	0.20	1.3	569	0.01	1.4
adaptive MS	38626	5155	0.13	0.9	443	0.01	1.2
adaptive SS	35567	5478	0.15	1.0	353	0.01	1.0
