# Supplementary material for: Finite Adaptation and Multistep Moves in the Metropolis-Hastings Algorithm for Variable Selection in Genome-Wide Association Analysis
Source: PLoS One. 2012 Nov 15;7(11):e49445. doi: 10.1371/journal.pone.0049445 (PMC3499564; doi:10.1371/journal.pone.0049445)
Supplement: Text S1 — Supplementary methods. (PDF) [file pone.0049445.s012.pdf]

Finite Adaptation and Multistep Moves in the Metropolis-Hastings Algorithm for Variable Selection in Genome-Wide Association Analysis

Supplementary Text S1

Tomi Peltola, Pekka Marttinen, and Aki Vehtari

Supplementary methods

Computation: conditional distributions

The conditional distributions for the Gibbs sampling are derived below. Note that ω is marginalized out analytically and need not enter the computations. The full posterior distribution is

$$p(\alpha, \boldsymbol{\eta}, \sigma^2, \boldsymbol{\tau}^2, \boldsymbol{\gamma}|\boldsymbol{y}) \propto p(\alpha)p(\boldsymbol{\eta}|\sigma^2, \boldsymbol{\tau}^2, \boldsymbol{\gamma})p(\sigma^2)p(\boldsymbol{\tau}^2)p(\boldsymbol{\gamma})p(\boldsymbol{y}|\alpha, \boldsymbol{\eta}, \sigma^2). \quad (1)$$

Step 1: $\boldsymbol{\tau}^2$

The components of $\boldsymbol{\tau}^2$ can be sampled independently with only the corresponding element of $\boldsymbol{\eta}$ affecting the sampling. For efficiency of the implementation, only τ_j^2 for j with $\gamma_j = 1$ are actually sampled in this step as the others do not affect the linear model. For others, the full conditional distribution is the prior and sampling is done just-in-time, if they are considered for addition to the model in the third step.

When $\gamma_j = 1$,

$$\begin{aligned} p(\tau_j^2|\alpha, \boldsymbol{\eta}, \sigma^2, \boldsymbol{\tau}_{-j}^2, \boldsymbol{\gamma}, \boldsymbol{y}) &\propto p(\tau_j^2)p(\eta_j|\sigma^2, \tau_j^2, \gamma_j) \quad (2)\\ &= \mathrm{Inv}-\chi^2(\tau_j^2|\nu_\tau, s_\tau^2)\mathrm{N}(\eta_j|0, \tau_j^2\sigma^2)\\ &\propto (\tau_j^2)^{-(\frac{\nu_\tau}{2}+1)}\exp(-0.5\nu_\tau s_\tau^2/\tau_j^2)(\tau_j^2)^{-\frac{1}{2}}\exp(-0.5\frac{\eta_j^2}{\sigma^2}/\tau_j^2)\\ &= (\tau_j^2)^{-(\frac{\nu_\tau+1}{2}+1)}\exp(-0.5(\nu_\tau s_\tau^2 + \frac{\eta_j^2}{\sigma^2})/\tau_j^2)\\ &\Rightarrow \mathrm{Inv}-\chi^2(\tau_j^2|\nu_\tau+1, \frac{\nu_\tau s_\tau^2 + \frac{\eta_j^2}{\sigma^2}}{\nu_\tau+1}) \end{aligned}$$

Step 2: α

$$\begin{aligned}
p(\alpha|\boldsymbol{\eta},\sigma^2,\boldsymbol{\tau}^2,\boldsymbol{\gamma},\boldsymbol{y}) &\propto p(\alpha)p(\boldsymbol{y}|\alpha,\boldsymbol{\eta},\sigma^2) \\
&= \mathrm{N}(\alpha|\mu,1)\mathrm{N}(\boldsymbol{y}|\boldsymbol{X}\alpha\boldsymbol{\eta},\sigma^2\boldsymbol{I}) \\
&\propto \exp(-\frac{1}{2}(\alpha-\mu)^2)\exp(-\frac{1}{2\sigma^2}(\boldsymbol{y}-\boldsymbol{X}\alpha\boldsymbol{\eta})^T(\boldsymbol{y}-\boldsymbol{X}\alpha\boldsymbol{\eta})) \\
&\propto \exp(-\frac{1}{2}(1+\frac{\boldsymbol{\eta}^T\boldsymbol{X}^T\boldsymbol{X}\boldsymbol{\eta}}{\sigma^2})(\alpha-(1+\frac{\boldsymbol{\eta}^T\boldsymbol{X}^T\boldsymbol{X}\boldsymbol{\eta}}{\sigma^2})^{-1}(\mu+\frac{\boldsymbol{y}^T\boldsymbol{X}\boldsymbol{\eta}}{\sigma^2}))^2) \\
&\Rightarrow \mathrm{N}(\alpha|(1+\frac{\boldsymbol{\eta}^T\boldsymbol{X}^T\boldsymbol{X}\boldsymbol{\eta}}{\sigma^2})^{-1}(\mu+\frac{\boldsymbol{y}^T\boldsymbol{X}\boldsymbol{\eta}}{\sigma^2}),(1+\frac{\boldsymbol{\eta}^T\boldsymbol{X}^T\boldsymbol{X}\boldsymbol{\eta}}{\sigma^2})^{-1}),
\end{aligned} \tag{3}$$

where $\boldsymbol{I}$ is an identity matrix.

Step 3: $\boldsymbol{\gamma}$

Step 3 is implemented as a Metropolis-Hastings step. The proposal algorithms are described in the main text. The required posterior probability of $\boldsymbol{\gamma}$ for the acceptance probability is derived next. $\boldsymbol{\eta}$ and σ^2 are integrated over analytically, so they need not be conditioned on in this step. $\boldsymbol{\eta}$ can be divided into to parts, one with all η_j with the corresponding $\gamma_j = 0$ and the other with all η_j with $\gamma_j = 1$. The former part does not affect the likelihood and the integral over that part is $\prod_{j:\gamma_j=0}\int\delta_0(\eta_j)d\eta_j = 1$. The latter part affects the likelihood and is denoted $\boldsymbol{\eta}_{\boldsymbol{\gamma}}$ below (corresponding part of $\boldsymbol{X}$ is denoted $\boldsymbol{X}_{\boldsymbol{\gamma}}$).

$$\begin{aligned}
p(\boldsymbol{\gamma}|\alpha,\boldsymbol{\tau}^2,\boldsymbol{y}) &= \int\int p(\boldsymbol{\gamma},\boldsymbol{\eta},\sigma^2|\alpha,\boldsymbol{\tau}^2,\boldsymbol{y})d\boldsymbol{\eta}d\sigma^2 \\
&\propto p(\boldsymbol{\gamma})\int\int p(\boldsymbol{\eta}_{\boldsymbol{\gamma}}|\sigma^2,\boldsymbol{\tau}^2)p(\sigma^2)p(\boldsymbol{y}|\alpha,\boldsymbol{\eta}_{\boldsymbol{\gamma}},\sigma^2)d\boldsymbol{\eta}_{\boldsymbol{\gamma}}d\sigma^2
\end{aligned} \tag{4}$$

First integrating over $\boldsymbol{\eta}_{\boldsymbol{\gamma}}$ gives

$$\int \mathrm{N}(\boldsymbol{\eta}_{\boldsymbol{\gamma}}|0,\sigma^2\Sigma_\tau)\mathrm{N}(\boldsymbol{y}|\boldsymbol{X}_{\boldsymbol{\gamma}}\alpha\boldsymbol{\eta}_{\boldsymbol{\gamma}},\sigma^2\boldsymbol{I})d\boldsymbol{\eta}_{\boldsymbol{\gamma}} = (2\pi)^{-\frac{n}{2}}|\Sigma_\tau|^{-\frac{1}{2}}|\Sigma_\tau^{-1}+\alpha^2\boldsymbol{X}_{\boldsymbol{\gamma}}^T\boldsymbol{X}_{\boldsymbol{\gamma}}|^{-\frac{1}{2}}(\sigma^2)^{-\frac{n}{2}}\exp(-\frac{1}{2\sigma^2}S^2), \tag{5}$$

where Σ_τ is a diagonal matrix of the τ_j^2s with j such that $\gamma_j = 1$ and $S^2 = \boldsymbol{y}^T\boldsymbol{y} - \alpha^2\boldsymbol{y}^T\boldsymbol{X}_{\boldsymbol{\gamma}}(\Sigma_\tau^{-1} + \alpha^2\boldsymbol{X}_{\boldsymbol{\gamma}}^T\boldsymbol{X}_{\boldsymbol{\gamma}})^{-1}\boldsymbol{X}_{\boldsymbol{\gamma}}^T\boldsymbol{y}$.

Then the integration over σ^2 gives

$$\begin{aligned}
&\int \mathrm{Inv}-\chi^2(\sigma^2|\nu_\sigma,s_\sigma^2)\int \mathrm{N}(\boldsymbol{\eta}_{\boldsymbol{\gamma}}|0,\sigma^2\Sigma_\tau)\mathrm{N}(\boldsymbol{y}|\boldsymbol{X}_{\boldsymbol{\gamma}}\alpha\boldsymbol{\eta}_{\boldsymbol{\gamma}},\sigma^2\boldsymbol{I})d\boldsymbol{\eta}_{\boldsymbol{\gamma}}d\sigma^2 \\
&= \frac{\Gamma(\frac{\nu_\sigma+n}{2})}{\Gamma(\frac{\nu_\sigma}{2})}\pi^{-\frac{n}{2}}(\nu_\sigma s_\sigma^2)^{\frac{\nu_\sigma}{2}}|\Sigma_\tau|^{-\frac{1}{2}}|\Sigma_\tau^{-1}+\alpha^2\boldsymbol{X}_{\boldsymbol{\gamma}}^T\boldsymbol{X}_{\boldsymbol{\gamma}}|^{-\frac{1}{2}}(\nu_\sigma s_\sigma^2+S^2)^{-\frac{\nu_\sigma+n}{2}},
\end{aligned} \tag{6}$$

where $\Gamma(\cdot)$ is the gamma function. Combining this with the $\boldsymbol{\gamma}$ prior gives the required posterior probability (up to a scale factor, which cancels out in the acceptance probability).

Step 4: σ^2

$\boldsymbol{\eta}$ is integrated over analytically as in the previous step (Equation 5). Combining the result with the $\text{Inv}-\chi^2$ prior of σ^2 gives the conditional distribution:

$$
\begin{aligned}
p(\sigma^2|\alpha, \boldsymbol{\tau}^2, \boldsymbol{\gamma}, \boldsymbol{y}) &\propto (\sigma^2)^{-\frac{\nu_\sigma}{2}} \exp(\frac{\nu_\sigma s_\sigma^2}{2\sigma^2})(\sigma^2)^{-\frac{n}{2}} \exp(-\frac{S^2}{2\sigma^2}) \\
&= (\sigma^2)^{-\frac{\nu_\sigma+n}{2}} \exp(\frac{\nu_\sigma s_\sigma^2 + S^2}{2\sigma^2}) \\
&\Rightarrow \text{Inv}-\chi^2(\sigma^2|\nu_\sigma + n, \frac{\nu_\sigma s_\sigma^2 + S^2}{\nu_\sigma + n})
\end{aligned}
\tag{7}
$$

Step 5: $\boldsymbol{\eta}$

Finally, the conditional distribution for $\boldsymbol{\eta}_{\boldsymbol{\gamma}}$ part of $\boldsymbol{\eta}$ is (the other part is constrained to $\mathbf{0}$)

$$
\begin{aligned}
p(\boldsymbol{\eta}_{\boldsymbol{\gamma}}|\alpha, \sigma^2, \boldsymbol{\tau}^2, \boldsymbol{\gamma}, \boldsymbol{y}) &\propto p(\boldsymbol{\eta}_{\boldsymbol{\gamma}}|\sigma^2, \boldsymbol{\tau}^2)p(\boldsymbol{y}|\alpha, \boldsymbol{\eta}_{\boldsymbol{\gamma}}, \sigma^2) \\
&= \text{N}(\boldsymbol{\eta}_{\boldsymbol{\gamma}}|0, \sigma^2\Sigma_\tau)\text{N}(\boldsymbol{y}|\boldsymbol{X}_{\boldsymbol{\gamma}}\alpha\boldsymbol{\eta}_{\boldsymbol{\gamma}}, \sigma^2\boldsymbol{I}) \\
&\propto \exp(-\frac{1}{2\sigma^2}\boldsymbol{\eta}_{\boldsymbol{\gamma}}^T\Sigma_\tau^{-1}\boldsymbol{\eta}_{\boldsymbol{\gamma}}) \exp(-\frac{1}{2\sigma^2}(\boldsymbol{y} - \boldsymbol{X}\alpha\boldsymbol{\eta})^T(\boldsymbol{y} - \boldsymbol{X}\alpha\boldsymbol{\eta})) \\
&\propto \exp(-\frac{1}{2\sigma^2}(\boldsymbol{\eta}_{\boldsymbol{\gamma}} - (\Sigma_\tau^{-1} + \alpha^2\boldsymbol{X}_{\boldsymbol{\gamma}}^T\boldsymbol{X}_{\boldsymbol{\gamma}})^{-1}\alpha\boldsymbol{X}_{\boldsymbol{\gamma}}^T\boldsymbol{y})^T(\Sigma_\tau^{-1} + \alpha^2\boldsymbol{X}_{\boldsymbol{\gamma}}^T\boldsymbol{X}_{\boldsymbol{\gamma}}) \\
&\quad (\boldsymbol{\eta}_{\boldsymbol{\gamma}} - (\Sigma_\tau^{-1} + \alpha^2\boldsymbol{X}_{\boldsymbol{\gamma}}^T\boldsymbol{X}_{\boldsymbol{\gamma}})^{-1}\alpha\boldsymbol{X}_{\boldsymbol{\gamma}}^T\boldsymbol{y})) \\
&\Rightarrow \text{N}(\boldsymbol{\eta}_{\boldsymbol{\gamma}}|(\Sigma_\tau^{-1} + \alpha^2\boldsymbol{X}_{\boldsymbol{\gamma}}^T\boldsymbol{X}_{\boldsymbol{\gamma}})^{-1}\alpha\boldsymbol{X}_{\boldsymbol{\gamma}}^T\boldsymbol{y}, \sigma^2(\Sigma_\tau^{-1} + \alpha^2\boldsymbol{X}_{\boldsymbol{\gamma}}^T\boldsymbol{X}_{\boldsymbol{\gamma}})^{-1}).
\end{aligned}
\tag{8}
$$

Move size proposal

Pasarica and Gelman [2010] give importance sampling estimators for the objective function. The multiple importance sampling estimator from B batches with L_b samples each has form

$$
\hat{h}(p|p_{B-1}, \dots, p_0) = \frac{\sum_{b=1}^{B}\sum_{l=1}^{L_b} ||\boldsymbol{\gamma}_{b,l} - \boldsymbol{\gamma}_{b,l}^*||^2 a(\boldsymbol{\gamma}_{b,l}, \boldsymbol{\gamma}_{b,l}^*) w_{p|p_{B-1},\dots,p_0}(\boldsymbol{\gamma}_{b,l}, \boldsymbol{\gamma}_{b,l}^*)}{\sum_{b=1}^{B}\sum_{l=1}^{L_b} w_{p|p_{B-1},\dots,p_0}(\boldsymbol{\gamma}_{b,l}, \boldsymbol{\gamma}_{b,l}^*)},
\tag{9}
$$

where the weights $w_{p|p_{B-1},\dots,p_0}(\boldsymbol{\gamma}, \boldsymbol{\gamma}^*) = \frac{q_p(\boldsymbol{\gamma}^*, k|\boldsymbol{\gamma})}{\sum_{b=1}^{B} L_b q_{p_{b-1}}(\boldsymbol{\gamma}^*, k|\boldsymbol{\gamma})}$. We note that is a form of adaptive multiple importance sampling [Cornuet et al., 2012], as the p_bs are formed sequentially based on all past samples by maximizing the objective.

Assuming the decomposition of $q_p(\boldsymbol{\gamma}^*, k|\boldsymbol{\gamma}) = q_p(k)q(\boldsymbol{\gamma}^*|k, \boldsymbol{\gamma})$ and that the latter factor is independent of

p and b (which will not actually hold exactly in our sampler; see below), the weights simplify to include only the $q_p(k)$ factor. Now, the estimator may be rewritten as

$$\hat{h}(p|p_{B-1},\ldots,p_0) = \frac{\sum_k k q_p(k) \frac{1}{\sum L_b q_{b-1}(k)} \sum_b \sum_l I(k = k_{b,l}) a(\boldsymbol{\gamma}_{b,l}, \boldsymbol{\gamma}^*_{b,l})}{\sum_k q_p(k) \frac{1}{\sum L_b q_{b-1}(k)} \sum_b \sum_l I(k = k_{b,l})}, \tag{10}$$

where $I(\cdot) = 1$ if the condition holds and zero otherwise. The sum in the numerator can be read as the move size times the probability of proposing it and accepting (mean acceptance rate), that is, the expected move size with parameter p. Note that computing $\hat{h}$ requires only tracking the number of proposals of move size k, the expected number of proposals of move size k and the sum of the acceptance probabilities of moves of size k for each k during the sampling. The maximization can be robustly performed by computing the objective function for a number of values of p in a grid (we have used 50 equally spaced grid points in $[0.01, 0.99]$).

Our proposed sampling scheme will update also $q(\boldsymbol{\gamma}^*|k,\boldsymbol{\gamma})$ after each batch as will described below. Thus, the Equation 10 will not hold exactly. However, it is used in this work because of its simplicity compared to accounting for the adaptation of $q(\boldsymbol{\gamma}^*|k,\boldsymbol{\gamma})$. Moreover, the adaptation of $q(\boldsymbol{\gamma}^*|k,\boldsymbol{\gamma})$ diminishes with the growing number of batches and the Equation 10 should accordingly tend to better estimates, if the adaptive period is long enough. Heuristics could be used to downweight the contribution of early batches (where the samples may also not be from the stationary distribution) to the adaptation of p, but this is not implemented here. Notably, this issue does not affect the validity of the sampling algorithm when finite adaptation is used as long as the final transition kernel is valid.

The proposed sampling scheme will use auxiliary variables in the sampling and may include a delayed rejection step. This requires modifications to the above formulas. Taking the auxiliary variable $\boldsymbol{\zeta}$ into account in the objective function gives

$$h(p) = \mathrm{E}_J[||\boldsymbol{\gamma} - \boldsymbol{\gamma}^*||^2] = \sum_{\boldsymbol{\gamma}} \sum_{\boldsymbol{\zeta}^*} \sum_{\boldsymbol{\gamma}^*} ||\boldsymbol{\gamma} - \boldsymbol{\gamma}^*||^2 \pi(\boldsymbol{\gamma}) q_p(\boldsymbol{\zeta}^*, k|\boldsymbol{\gamma}) q(\boldsymbol{\gamma}^*|\boldsymbol{\zeta}^*, \boldsymbol{\gamma}) a(\boldsymbol{\gamma}; \boldsymbol{\zeta}^*, \boldsymbol{\gamma}^*, \boldsymbol{\zeta}), \tag{11}$$

where there is no need to sum over $\boldsymbol{\zeta}$ as it is deterministic given $\boldsymbol{\zeta}^*$ and $\boldsymbol{\gamma}$. With the factorization $q_p(\boldsymbol{\zeta}^*, k|\boldsymbol{\gamma}) q(\boldsymbol{\gamma}^*|\boldsymbol{\zeta}^*, \boldsymbol{\gamma}) = q_p(k|\boldsymbol{\gamma}) q(\zeta^*_{1:k}|k, \boldsymbol{\gamma}) q(\boldsymbol{\gamma}^*|\boldsymbol{\zeta}^*, \boldsymbol{\gamma})$, Equation 10 holds also when the sampling utilizes auxiliary variables.

With delayed rejection move the the objective function can be written as

$$h(p) = \mathrm{E}_J[||\boldsymbol{\gamma} - \boldsymbol{\gamma}'^*||^2] = \sum_{\boldsymbol{\gamma}} \sum_{\boldsymbol{\gamma}^*} \sum_{\boldsymbol{\gamma}'} \sum_{\boldsymbol{\gamma}'^*} ||\boldsymbol{\gamma} - \boldsymbol{\gamma}'^*||^2 \pi(\boldsymbol{\gamma}) q_p(\boldsymbol{\gamma}^*, \boldsymbol{\gamma}', k|\boldsymbol{\gamma}) q(\boldsymbol{\gamma}'^*|\boldsymbol{\gamma}^*, \boldsymbol{\gamma}', \boldsymbol{\gamma}), \tag{12}$$

where $q_p q$ is written using the view of delayed rejection, where both proposals ($\boldsymbol{\gamma}^*$ first and $\boldsymbol{\gamma}'$ second) are gen-

erated by q_p and then one is chosen in q as the new state $\boldsymbol{\gamma}'^*$ [Storvik, 2011]. Again $q_p(\boldsymbol{\gamma}^*, \boldsymbol{\gamma}', k|\boldsymbol{\gamma})q(\boldsymbol{\gamma}'^*|\boldsymbol{\gamma}^*, \boldsymbol{\gamma}', \boldsymbol{\gamma})$ factorizes into $q_p(k|\boldsymbol{\gamma})$ and a part which is independent of p and this may be used to simplify computation in the multiple importance sampling. However, now the acceptance probability is always one and moves with sampled move size being k may have $||\boldsymbol{\gamma} - \boldsymbol{\gamma}'^*||^2 < k$. Thus, instead of Equation 10, the simplified estimator may be written as

$$\hat{h}(p|p_{B-1}, \ldots, p_0) = \frac{\sum_k \frac{q_p(k)}{\sum L_b q_{b-1}(k)} \sum_b \sum_l I(k = k_{b,l}) ||\boldsymbol{\gamma}_{b,l} - \boldsymbol{\gamma}'^*_{b,l}||^2}{\sum_k \frac{q_p(k)}{\sum L_b q_{b-1}(k)} \sum_b \sum_l I(k = k_{b,l})}. \tag{13}$$

It is possible to write the non-DR version also without the acceptance probability (identically to above with $\boldsymbol{\gamma}'^*_{b,l} = \boldsymbol{\gamma}_{b,l}$ if move is rejected), but using the knowledge of the acceptance probabilities could be expected to give more stable estimates.

Note on the sequential sampling

The proposal distribution $q_1(\boldsymbol{\gamma}^*|\boldsymbol{\gamma})$ was augmented with auxiliary variables fixing the order in which the variables have been proposed as the proposal probability depends on this order. Alternatively, one may sum over the different orderings to get rid of this dependency. The latter can be shown to have smaller asymptotic variance for MCMC estimates (see Storvik [2011]), but may be slow to compute. For example, for k additions the sum has factorial of k terms and naive summing would be inefficient already with moderate k (with $k = 10$ there are more than 3.6 million terms).

We note that separating the sampling of additions and removals would allow considering the sequential sampling of each as a biased urn scheme (e.g., the urn for additions contains all variables, which are not in the model and has not been yet proposed to be added in this proposal). The scheme can be recognised as a special case of the Wallenius' noncentral hypergeometric distribution, for which summing over the orderings would be possible, for example, with one dimensional numerical integration, where the complexity of the integrand scales linearly in k [Fog, 2008]. However, this is not pursued further here.

Delayed rejection and the deterministic procedure of h_1 and h_2

The second proposal is made from a discrete distribution with 2^k states, where the proposal probabilities are chosen such that the proposal will always be accepted (with positive probability for proposing the old state $\boldsymbol{\gamma}$). In order to make all factors cancel in a_2, we choose $q_2(\boldsymbol{\gamma}'|\boldsymbol{\gamma}, \boldsymbol{\zeta}^*) = \pi(\boldsymbol{\gamma}')q_1(\boldsymbol{\gamma}'^*, \boldsymbol{\zeta}'^*, k|\boldsymbol{\gamma}')(1 - a_1(\boldsymbol{\gamma}'; \boldsymbol{\zeta}'^*, \boldsymbol{\gamma}'^*, \boldsymbol{\zeta}'))/Z$, that is, the product of the posterior probability of the proposed model, the probability of making a first proposal from the proposed model with updates to the variables specified by $\boldsymbol{\zeta}^*$ and the

probability of rejecting the hypothetical first proposal. Z normalizes the distribution over the 2^k models. It is required that $q_2(\boldsymbol{\gamma}'|\boldsymbol{\gamma},\boldsymbol{\zeta}^*) = q_2(\boldsymbol{\gamma}'|\boldsymbol{\gamma}',\boldsymbol{\zeta}'^*)$, so that the proposal probabilities and Z do not depend on which of the 2^k pairs $(\boldsymbol{\gamma},\boldsymbol{\zeta}^*)$ q_2 is conditioned on.

To this end, we need to have correspondence between the pairs. The following algorithm is used to determine $\boldsymbol{\zeta}'^*$ for given $\boldsymbol{\gamma}'$ and $(\boldsymbol{\gamma},\boldsymbol{\zeta}^*)$ in q_2 and h_2 (i.e., $h_2(\boldsymbol{\zeta}'^*|\boldsymbol{\gamma}',\boldsymbol{\gamma},\boldsymbol{\zeta}^*) = 1$ when $\boldsymbol{\zeta}'^*$ follows from this algorithm and zero otherwise; similarly also h_1):

1. Form an intermediate $\boldsymbol{\zeta}^b$: 1) set $\boldsymbol{\zeta}^b = \boldsymbol{\zeta}^*$, 2) reverse the order of removals in $\boldsymbol{\zeta}^b$, 3) set all operations in $\boldsymbol{\zeta}^b$ to additions.

2. Form $\boldsymbol{\zeta}'^*$: 1) set $\boldsymbol{\zeta}'^* = \boldsymbol{\zeta}^b$, 2) convert such elements of $\boldsymbol{\zeta}'^*$ to removals, which are required for it to be a valid first stage proposal from state $\boldsymbol{\gamma}'$, 3) reverse the order of removals.

Notice that performing the step 1 for $\boldsymbol{\zeta}'^*$ leads to the same $\boldsymbol{\zeta}^b$ as using $\boldsymbol{\zeta}^*$. Also, if in step 2.2 $\boldsymbol{\gamma}'$ is replaced with $\boldsymbol{\gamma}$, then $\boldsymbol{\zeta}'^* = \boldsymbol{\zeta}^*$. Further, as all permutations of the order of the elements in $\boldsymbol{\zeta}^*$ lead to different $\boldsymbol{\zeta}^b$, there is only one $\boldsymbol{\zeta}'^*$ corresponding to $\boldsymbol{\zeta}^*$.

It would be equally valid to keep the original order and only swap the operations (additions and removals) appropriately. However, if we have sampled $\boldsymbol{\zeta} = (\text{add } 1, \text{add } 2)$, then we may expect that a reverse move with $(\text{remove } 2, \text{remove } 1)$ has larger proposal probability than $(\text{remove } 1, \text{remove } 2)$ on average. Hence, reversing the order makes sense with regard to maximizing the proposal probabilities.

Exhaustive computation of a set of models through updates to the Cholesky decomposition

Details on the computation of the 2^k models in the delayed rejection step are given below. First, the formula for the marginal likelihood of the linear regression model is given and its computation via Cholesky decomposition briefly described. We briefly review the Cholesky update operations required and refer to Clark [1981] for more details on the traversal of the 2^k models in $O(2^k k)$ computational complexity.

The marginal likelihood after integrating analytically out the parameters $\boldsymbol{\beta}$ and σ^2 is following (where $\tau_\alpha^2 = \alpha^2\tau^2$):

$$
\begin{aligned}
p(\boldsymbol{y}|\boldsymbol{X},\tau_\alpha^2,\boldsymbol{\gamma}) &= \int p(\boldsymbol{\beta}|\sigma^2,\tau_\alpha^2,\boldsymbol{\gamma})p(\sigma^2)p(\boldsymbol{y}|\boldsymbol{\beta},\sigma^2,\boldsymbol{X})d\boldsymbol{\beta}d\sigma^2 \\
&= \pi^{-n/2}|\boldsymbol{\Sigma}|^{-1/2}|\boldsymbol{X}_{\boldsymbol{\gamma}}^T\boldsymbol{X}_{\boldsymbol{\gamma}} + \boldsymbol{\Sigma}^{-1}|^{-1/2}\frac{\Gamma((\nu_\sigma+n)/2)}{\Gamma(\nu_\sigma/2)}(\nu_\sigma s_\sigma^2)^{\nu_\sigma/2}(\nu_\sigma s_\sigma^2 + S^2)^{-(\nu_\sigma+n)/2},
\end{aligned}
$$

where $S^2 = \boldsymbol{y}^T\boldsymbol{y} - \boldsymbol{y}^T\boldsymbol{X}_{\boldsymbol{\gamma}}(\boldsymbol{X}_{\boldsymbol{\gamma}}^T\boldsymbol{X}_{\boldsymbol{\gamma}} + \boldsymbol{\Sigma}^{-1})^{-1}\boldsymbol{X}_{\boldsymbol{\gamma}}^T\boldsymbol{y}$ and $\boldsymbol{\Sigma}$ is the prior covariance matrix of $\boldsymbol{\beta}_{\boldsymbol{\gamma}}$ (subscripting with $\boldsymbol{\gamma}$ refers to taking only such elements for which $\gamma_j = 1$).

Let $A = \boldsymbol{X}_{\boldsymbol{\gamma}}^T \boldsymbol{X}_{\boldsymbol{\gamma}} + \boldsymbol{\Sigma}^{-1}$ and $b = \boldsymbol{X}_{\boldsymbol{\gamma}}^T \boldsymbol{y}$. The Cholesky factor of A is an upper triangular matrix U, such that $A = U^T U$. Computing S_{yy} amounts to computing $\boldsymbol{y}^T \boldsymbol{y} - v^T v$, where v is solved from $U^T v = b$. Note that the determinant $|A| = |U|^2$, where $|U|$ can be computed as the product of the diagonal elements of U (3.11, Dongarra et al. [1979]). The computational complexity of the Cholesky decomposition is $O(q^3)$, where q is the number of variables [Dongarra et al., 1979].

Taking A_1 to be the leading submatrix (upperleft-most square submatrix) of A, then the corresponding part of U, U_1, is its Cholesky factor (i.e., $A_1 = U_1^T U_1$) (8, Dongarra et al. [1979]). Consequently, computing $\boldsymbol{y}^T \boldsymbol{y} - v_{1:r}^T v_{1:r}$, where $v_{1:r}$ is a subvector with the r first elements of v allows the computation of the S_{yy} for a model with only the r first variables (the solution to $U^T v = b$ for the first r elements does not depend on the rest as U^T is lower triangular).

This enables introducing new variables to the model by adding them to new positions to the end of the Cholesky decomposition. The old values will not change and only the new part needs to be computed. In fact, adding a new variable corresponds to running one new iteration of the algorithm, which has complexity $O(q^2)$ (see the factorization algorithm at 3.10 of Dongarra et al. [1979]), after the computation of the new covariances. Removing variables from the model amounts to moving them to the last positions and then dropping their contribution to the likelihood. This requires being able to do updates to the Cholesky decomposition when positions of variables are swapped. Swapping two adjacent variables can be done with a single Givens rotation, where multiplying the rotation into the matrix has complexity $O(q)$ (10, Dongarra et al. [1979]). Moving a variable from an internal position to last requires $q - i$ rotations, where i is the position of the variable.

These operations facilitate algorithms for computing the 2^k models with only swaps of positions of adjacent variables. With the number of swaps being $2^k - k - 1$ [Clark, 1981] the computational complexity for the algorithm is $O(2^k k)$, assuming as a starting point a full Cholesky decomposition with the k variables at the last positions (this may require some additional Cholesky updates when the first, rejected proposal in the delayed rejection algorithm involves removals). Now, traversing all of the models amounts to visiting all subsets of the set $\{1, 2, \ldots, k\}$ in an appropriate order. Clark [1981] gives one such algorithm.

Kohn-Smith-Chan and Nott-Kohn algorithms

We may write some of the alternative schemes proposed in literature to a similar form as in the samplers in the main text by selecting q_1 and h_1 appropriately. We assume that the move (or block) size k is fixed as in the standard implementations of these algorithms. We take $q_1(\boldsymbol{\gamma}^*, \boldsymbol{\zeta}^* | \boldsymbol{\gamma}, k) = q(\boldsymbol{\zeta}^* | \boldsymbol{\gamma}, k) q(\boldsymbol{\gamma}^* | \boldsymbol{\zeta}^*, \boldsymbol{\gamma})$ with $q(\boldsymbol{\zeta}^* | \boldsymbol{\gamma}, k) \propto 1$ (i.e., given move size, select the variables to update uniformly) and $h(\boldsymbol{\zeta} | \boldsymbol{\zeta}^*) = 1$ if $\boldsymbol{\zeta} = \boldsymbol{\zeta}^*$

and zero otherwise for this purpose. The uniform sampling of $\boldsymbol{\zeta}^*$ gives "random scan" versions of Gibbs-like algorithms, which have similar definition of iteration to the algorithms proposed in the main text.

Random scan version of Kohn-Smith-Chan (KSC) sampler [Kohn et al., 2001] takes $q(\boldsymbol{\gamma}^*|\boldsymbol{\zeta}^*, \boldsymbol{\gamma}, k) = p(\boldsymbol{\gamma}_{\boldsymbol{\zeta}^*}|\boldsymbol{\gamma}_{-\boldsymbol{\zeta}^*})$, that is, it proposes a model from the 2^k models available as permutations of the k selected variables with the sampling probabilities proportional to the prior probabilities. Random scan version of the Nott-Kohn (NK) sampler [Nott and Kohn, 2005] uses an adaptive distribution for $q(\boldsymbol{\gamma}^*|\boldsymbol{\zeta}^*, \boldsymbol{\gamma})$ (here, the marginal inclusion probabilities adapted similar to the proposed algorithms in the main text, although Nott and Kohn [2005] advocate the use of an empirical covariance matrix of γ to account for correlations; however, in applications to datasets in the scale considered in this work estimating the covariance matrix is not feasible unless some additional structure would be imposed on it).

The NK sampler and the tuned sampler described in the main text are similar. An off-diagonal element in the transition matrix of the single-step NK Markov chain is

$$P_{NK}(\boldsymbol{\gamma}, \boldsymbol{\gamma}^*) = \begin{cases} 0 & \text{if } ||\boldsymbol{\gamma} - \boldsymbol{\gamma}^*|| > 1 \\ \frac{1}{m}\pi_j \min(1, \frac{\pi(\boldsymbol{\gamma}^*)(1-\pi_j)}{\pi(\boldsymbol{\gamma})\pi_j}) & \text{if } ||\boldsymbol{\gamma} - \boldsymbol{\gamma}^*|| \leq 1 \text{ and } \gamma_j = 0 \\ \frac{1}{m}(1-\pi_j) \min(1, \frac{\pi(\boldsymbol{\gamma}^*)\pi_j}{\pi(\boldsymbol{\gamma})(1-\pi_j)}) & \text{if } ||\boldsymbol{\gamma} - \boldsymbol{\gamma}^*|| \leq 1 \text{ and } \gamma_j = 1, \end{cases} \tag{14}$$

where m is the number of variables, $\pi(\cdot)$ is the posterior probability of a model and π_j is the proposal probability for jth variable (adapted to the marginal posterior inclusion probability).

For the sampler described in the main text

$$P(\boldsymbol{\gamma}, \boldsymbol{\gamma}^*) = \begin{cases} 0 & \text{if } ||\boldsymbol{\gamma} - \boldsymbol{\gamma}^*|| > 1 \\ 0.5\frac{\pi_j}{Z_a} \min(1, \frac{\pi(\boldsymbol{\gamma}^*)(1-\pi_j)/Z'_r}{\pi(\boldsymbol{\gamma})\pi_j/Z_a}) & \text{if } ||\boldsymbol{\gamma} - \boldsymbol{\gamma}^*|| \leq 1 \text{ and } \gamma_j = 0 \\ 0.5\frac{(1-\pi_j)}{Z_r} \min(1, \frac{\pi(\boldsymbol{\gamma}^*)\pi_j/Z'_a}{\pi(\boldsymbol{\gamma})(1-\pi_j)/Z_r}) & \text{if } ||\boldsymbol{\gamma} - \boldsymbol{\gamma}^*|| \leq 1 \text{ and } \gamma_j = 1, \end{cases} \tag{15}$$

where the 0.5 factors come from taking the move to be an addition or removal and assuming that $\boldsymbol{\gamma}$ is not the empty model or the full model. The normalization constants in the acceptance probability will often approximately cancel in the equilibrium phase of the sampling, which leaves the difference in the transition matrices to the selection probabilities ($2Z$ vs m). Now, probably $2Z < m$, meaning that with these assumptions the off-diagonal elements of the latter sampler are larger. However, it should be noted that an essential feature of the NK sampler is that is has diagonal mass from such moves that do not require likelihood evaluations at all and are thus fast ($\boldsymbol{\gamma}$ has $\gamma_j = 1$ (or 0) and the proposal is to keep $\gamma_j = 1$ (0); then $\boldsymbol{\gamma} = \boldsymbol{\gamma}^*$ and the move is always accepted but the chain remains still).

References

Clark, M. R. B. (1981), "Algorithm AS 163: A Givens Algorithm for Moving from One Linear Model to Another Without Going Back to the Data," *J Roy Stat Soc C*, 30, 198–203.

Cornuet, J.-M., Marin, J.-M., Mira, A., and Robert, C. P. (2012), "Adaptive multiple importance sampling," *Scand J Stat*, Early View (online).

Dongarra, J. J., Moler, C. B., Bunch, J. R., and Stewart, G. W. (1979), *LINPACK Users' Guide*, SIAM.

Fog, A. (2008), "Calculation methods for Wallenius' noncentral hypergeometric distribution," *Commun Stat Simulat*, 37, 258–273.

Kohn, R., Smith, M., and Chan, D. (2001), "Nonparametric regression using linear combinations of basis functions," *Stat Comput*, 11, 313–322.

Nott, D. J. and Kohn, R. (2005), "Adaptive sampling for Bayesian variable selection," *Biometrika*, 92, 747–763.

Pasarica, C. and Gelman, A. (2010), "Adaptively scaling the Metropolis algorithm using expected squared jumped distance," *Stat Sinica*, 20, 343–364.

Storvik, G. (2011), "On the flexibility of Metropolis-Hastings acceptance probabilities in auxiliary variable proposal generation," *Scand J Stat*, 38, 342–358.
