# Supplementary material for: Finite Adaptation and Multistep Moves in the Metropolis-Hastings Algorithm for Variable Selection in Genome-Wide Association Analysis
Source: PLoS One. 2012 Nov 15;7(11):e49445. doi: 10.1371/journal.pone.0049445 (PMC3499564; doi:10.1371/journal.pone.0049445)
Supplement: Text S2 — Prior parameters for the simulation, HDL-C and LDL-C models. (PDF) [file pone.0049445.s013.pdf]

Finite Adaptation and Multistep Moves in the Metropolis-Hastings Algorithm for Variable Selection in Genome-Wide Association Analysis

Supplementary Text S2

Tomi Peltola, Pekka Marttinen, and Aki Vehtari

Prior parameters

The following parameters were chosen for the simulation runs. $\nu_\sigma = 1$ and s_σ^2 was set so that the mode of the implied proportion of variance explained "density" was 0.2 or 0.5 according to the simulation configuration (see Peltola et al. [2012]). $\mu = 1$, $\nu_\tau = 5$ and $s_\tau^2 = 0.005/0.012/0.0025/0.006$ (30 causal SNPs, $H^2 = 0.2$ / 30 causal SNPs, $H^2 = 0.5$ / 100 causal SNPs, $H^2 = 0.2$ / 100 causal SNPs, $H^2 = 0.5$). a_ω and b_ω were set so that the mean of the expected number of associations was 30 or 100 according to the simulation configuration with variances 900 and 9000, respectively (see Kohn et al. [2001]; Peltola et al. [2012]).

The results of a large meta-analysis [Teslovich et al., 2010], which included HDL-C and LDL-C analyses, were used as prior knowledge to guide in setting the parameters of HDL-C and LDL-C runs. The $\nu_\sigma = 1$ and s_σ^2 was set so that the mode of the implied proportion of variance explained "density" was 0.41 for HDL-C and 0.30 for LDL-C (note that this includes the expected effect of fixed covariates also). $\mu = 0$, $\nu_\tau = 1$ and $s_\tau^2 = 0.02$. a_ω and b_ω were set so that the mean of the expected number of associations was 47 for HDL-C and 37 for LDL-C with variances 1600 and 900, respectively.

References

Kohn, R., Smith, M., and Chan, D. (2001), "Nonparametric regression using linear combinations of basis functions," *Stat Comput*, 11, 313–322.

Peltola, T., Marttinen, P., Jula, A., Salomaa, V., Perola, M., and Vehtari, A. (2012), "Bayesian variable selection in searching for additive and dominant effects in genome-wide data," *PLoS ONE*, 7, e29115.

Teslovich, T. M., Musunuru, K., Smith, A. V., Edmondson, A. C., Stylianou, I. M., Koseki, M., Pirruccello,

J. P., Ripatti, S., Chasman, D. I., and Willer, C. J. e. a. (2010), "Biological, clinical and population relevance of 95 loci for blood lipids," *Nature*, 466, 707–713.
